# 2-Amino-1,3-benzothiazole: Endo *N*-Alkylation with α-Iodo Methyl Ketones Followed by Cyclization

**DOI:** 10.3390/molecules28052093

**Published:** 2023-02-23

**Authors:** Ivan A. Dorofeev, Larisa V. Zhilitskaya, Nina O. Yarosh, Bagrat A. Shainyan

**Affiliations:** A. E. Favorsky Irkutsk Institute of Chemistry, Siberian Division of the Russian Academy of Sciences, 1 Favorsky Street, 664033 Irkutsk, Russia

**Keywords:** 2-amino-1,3-benzothiazole, iodoacetone, 1,3-diiodoacetone, aromatic, heteroaromatic α-iodo methyl ketones, N-alkylation, intramolecular cyclization, iodide and triiodide salts

## Abstract

Reactions of 2-amino-1,3-benzothiazole with aliphatic, aromatic and heteroaromatic α-iodoketones in the absence of bases or catalysts have been studied. The reaction proceeds by N-alkylation of the endocyclic nitrogen atom followed by intramolecular dehydrative cyclization. The regioselectivity is explained and the mechanism of the reaction is proposed. A number of new linear and cyclic iodide and triiodide benzothiazolium salts have been obtained and their structure proved by NMR and UV spectroscopy.

## 1. Introduction

The synthesis of molecular hybrids containing structural fragments showing useful properties is one of the main goals in modern organic chemistry. Such hybrids are successfully applied in the syntheses of drugs, for the creation of new materials for advanced technologies, and for the development of organic syntheses. An important example is the derivatives of 2-aminothiazole. They are used as precursors of biologically active compounds in medicine and agriculture [1,2,3,4,5], are valuable components of matrices of inert coatings in industry [6], are used to disperse dyes [7], are adsorbents of heavy metals [8], and are used as fluorescent sensors for the detection of metals [9].

α-Chlorinated and brominated ketones are known to react with *S*,*N*-heterocycles via aminothiazolium salts as key intermediates, whose subsequent intramolecular cyclization by heating in ethanol results in condensed heterocyclic compounds with bridged nitrogen atoms [10,11,12,13,14,15,16,17,18,19,20,21,22,23,24]. The interest in such structures is due to their pharmacological potential. Thus, the imidazo[2,1-*b*]thiazole motif is proposed as a universal synthon for the design of molecules with antiviral [2], antidiabetic [12], antitumor [13,14], antifungal [15], antioxidant [15], and anti-inflammatory activity [16]. Some derivatives based on the imidazo[2,1-*b*]thiazole system have become commercial pharmaceuticals. Among them is an anthelmintic and immunomodulator Levamisol (trademark Ergamisol).

Prior to our studies, there were no data in the literature on the alkylation of aminothiazoles with α-iodoketones in the absence of bases or catalysts [25]. The synthesis of the salts of heterocyclic compounds is topical because of the growing interest in these compounds possessing a wide range of applications, both in science and in technique [26,27,28]. With this in mind, the goal of this work was to investigate the reactions of 2-amino-1,3-benzothiazole with aliphatic, aromatic, and heteroaromatic α-iodoketones, assuming that the high reactivity of the C–I bond in α-iodoketones might allow us to use them successfully for the synthesis of 2-amino-1,3-benzothiazole derivatives, and condensed structures on their basis, under mild conditions. Another goal of the present work was to compare the regioselectivity of *N*-alkylation—on the endo or exocyclic nitrogen atom of the substrate.

## 2. Results and Discussion

**Scheme 1:**
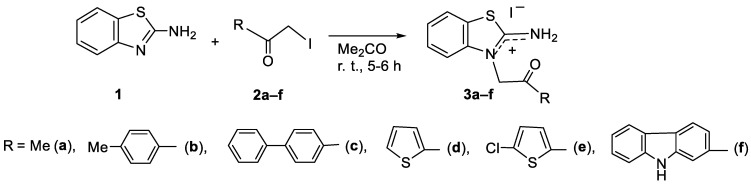
The reactions of 2-amino-1,3-benzothiazole **1** with α-iodoketones **2a**–**f**.

The reactions of 2-amino-1,3-benzothiazole **1** with α-iodoketones **2a**–**f** were carried out at room temperature in acetone in the absence of bases or catalysts. The reaction proceeds by *N*-alkylation of the endocyclic nitrogen atom with the formation of 2-amino-1,3-benzothiazolium iodides **3a**–**f**, which precipitated from the reaction mixture and could be isolated in pure form in a 51−74% yield (Scheme 1).

Similar reactions with α-chloro or bromoketones proceed more slowly and require heating [10,11,12,13,14,15,16,17,18,19,20,21,22,23,24]. An exception is microwave activation, as was recently exemplified by a highly efficient microwave-assisted procedure (mw, 100 °C, 12–15 min) for the synthesis of benzo[*d*]imidazo[2,1-*b*]thiazoles and N-alkylated 2-aminobenzo[*d*]oxazoles in aqueous media in up to 95% yields [29].

**Scheme 2:**
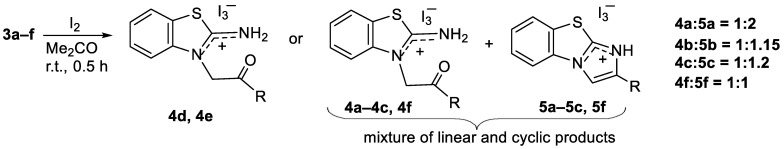
The reactions of salts **3a**–**f** with elemental iodine.

Under the conditions of Scheme 1, salts **3a**–**f** react with elemental iodine to give 2-amino-1,3-benzothiazolium triiodides **4d**,**e** isolated in a 90% yield (Scheme 2). The latter compounds combine the biological activity of elemental iodine with the organic component and show a wider spectrum of pharmacological properties. Drugs based on 1,3-diethylbenzimidazolium triiodides are known in the literature [27]. Unfortunately, we failed to isolate other linear triiodides in pure form. When reacting with elemental iodine, iodides **3a**–**c**,**f** undergo intramolecular cyclization to give an inseparable mixture of linear **4a**–**c**,**f** and cyclic triiodides **5a**–**c**,**f**. This was witnessed by the presence of the ^1^H and ^13^C NMR (Supplementary Materials) signals in the spectra of these compounds at 5.7–6.4 and 52.9–54.7 ppm, corresponding to the methylene groups in the acyclic products, as well as by the signals at 8.4–9.7 and 114.2–116.4 ppm of the =CH group of the imidazole moiety in the annulated products. The thienyl derivatives were the most resistant to cyclization, which was proved by calculations (vide infra).

As follows from Scheme 2, the ratio of products 4:5 decreases in the order R = Me > 4-tolyl ≥ 4-diphenyl > 2-carbazolyl, implicitly indicating the same order of the rate of cyclization.

**Scheme 3:**
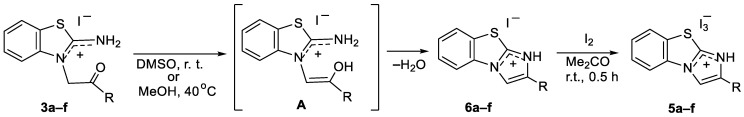
Cyclization of iodides **3a**–**f** to **6a**–**f** and conversion of the latter to triiodides **5a**–**f**.

Iodides **3a**–**f**, when stored in DMSO solution or with slight heating in MeOH, undergo cyclization with the formation of iodides **6a**–**f**. The formation of the benzo[*d*]imidazo[2,1-*b*]thiazolium structure is proved by the disappearance of the methylene CH_2_C(O) proton signal in the ^1^H NMR spectrum at 5.4–6.4 ppm, typical for linear 2-aminobenzothiazolium iodides **3a**–**f**, and the appearance of the vinyl proton signal of **5a**–**f** at 8.4–9.0 ppm. Similar to salts **3** in Scheme 1, salts **6a**–**f** are converted to benzo[*d*]imidazo[2,1-*b*]thiazolium triiodides **5a**–**f** (Scheme 3).

The cyclization is fast for R = Me, whereas for R = Ph and heteroaryl, it needs to stay in DMSO solution for several weeks. To get a deeper insight, we have calculated the reaction of cyclization **3** → **5** by the B3LYP/DGDZVP method with full geometry optimization in polar medium using a PCM model with DMSO as the solvent for R = Me, Ph, 2-thienyl, and 2-carbazolyl. The results (in kcal/mol) are shown in the table.


**R**

**Δ*E*° = *E*°(5 + H_2_O) − *E*°(3)**

**Δ*G*° = *G*°(5 + H_2_O) − *G*°(3)**
Me+4.0−9.2Ph+5.2−10.82-thienyl+6.3−3.82-carbazolyl+5.9−7.5

As evident from the table, for all R the reaction is endothermic but exergonic, that is, thermodynamically allowed. The cyclization of salt **3a** (R = Me) is the least endothermic and, according to Hammond’s postulate, has the lowest activation barrier. The calculation of salts **3** showed that the planes of the benzoyl or hetaroyl groups are nearly perpendicular to the benzothiazole ring. Since cyclization proceeds via the enol intermediate **A** followed by intramolecular dehydration (Scheme 2), the observed retardation of cyclization for R = Ph, hetaryl, is, apparently, due to the energy loss from the rotation of bulky R groups to form planar enol structure.

The calculations also allowed for explanation of the predominant endo *N*-alkylation of compound **1**. The HOMO of molecule **1** is nearly equally localized on the endo and exo nitrogen atoms, which allows one to assume that the formation of highly polar products **3** should be charge-controlled. The Mulliken charges on the endo and exo nitrogen atoms are equal to −0.260 and −0.720, respectively, which at first glance indicates a preferable exo *N*-alkylation. However, if one considers the charges with summed hydrogens, the values are −0.260 and +0.420, respectively, clearly showing the preference of the endo *N*-alkylation.

The question of regioselectivity of alkylation is of particular interest. In most of the referenced works [10,11,12,13,14,15,16,17,18,19,20,21,29], *N*-alkylation is reported to take place at the endocyclic nitrogen atom of the thiazole ring. Only in the recent works of Egyptian authors [22,23,24] is it stated that the imidazo[2,1-*b*]thiazole skeleton is formed by *N*-alkylation of the exocyclic primary amino group. However, in neither of these citations was there any experimental evidence of the structure of the formed product. The only such evidence was given in our recent work [25] by isolation of the imidazothiazolium iodide salts, whose structure was confirmed by the {^1^H–^15^N} HMBC experiments. In particular, a large upfield shift of the endocyclic ^15^N signal with respect to that in the starting aminothiazole was observed (by ~90 ppm, from −138.5 to −227.7 ppm), and a much smaller downfield shift of the exocyclic NH_2_ signal (by ~11 ppm, from −301.3 to −290.1 ppm). In the cyclization product, the signals of N_endo_ and N_exo_ appear at −174.1 and −219.4 ppm, respectively [25]. Along with the results of theoretical calculations, this allows us to conclude that alkylation occurs at the endocyclic nitrogen atom.

**Scheme 4:**
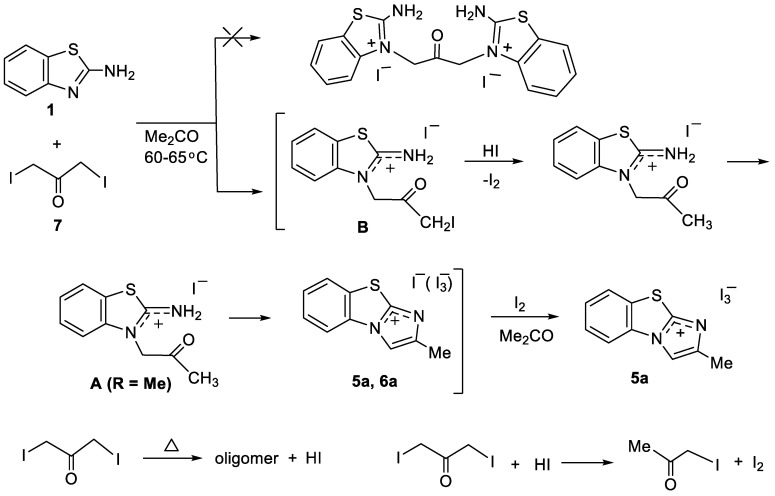
Reaction of 2-aminobenzothiazole **1** with 1,3-diiodoacetone **7**.

The presence of two iodomethyl groups in the molecule of 1,3-diiodoacetone might expand the synthetic utility of the process by allowing the second molecule of compound **1** to be involved in the reaction. This could result in bis-derivatives of compound **1**, by analogy with our recent work [30,31]. However, according to the NMR spectroscopy data of the reaction mixture, a mixture of the products of alkylation, cyclization and oligomerization was formed. The signals of the products of alkylation and cyclization coincide with those of compounds **3a** and **5a** obtained by alkylation of compound **1** with iodoacetone. The formation of the products of oligomerization is, probably, due to the heating of the reaction mixture to 65 °C in light, resulting in the appearance of HI [32]. Hydrogen iodide, in turn, can reduce the iodomethyl groups either in diiodoacetone **7**, or in intermediate **B** (Scheme 4), as proved by the appearance of the methyl group signal at 2.2 ppm in the ^1^H NMR spectrum. The formed elemental iodine initiated the intramolecular cyclization of the product of N-alkylation **3a,** converting it to **5a**. It was impossible to separate salts **3a**, **6a** and **5a**; therefore, the mixture was treated with elemental iodine to obtain the product of intramolecular condensation **5a** (Scheme 3), whose characteristics were identical to those obtained by the reaction with iodoacetone in Scheme 2.

All products were obtained as light-yellow or dark-red solids, and their structure was proved by NMR and UV spectroscopy and elemental analysis. Unfortunately, we were unable to isolate single crystals suitable for X-ray analysis, probably because of its insolubility in most of the conventional organic solvents. Compounds **4d**,**e** and **5a**–**f** combine different pharmacophores in one molecule in conjunction with iodine anions and, therefore, are potentially biologically active compounds. The evaluation of biological activity of organic compounds is ongoing.

## 3. Experimental Section

### 3.1. General

Melting points were determined on a Micro-Hot-Stage PolyTherm A. Elemental analysis was carried out on a CHNS analyzer Thermo Scientific Flash 2000. The iodine was determined by mercurimetric titration. The reactions were monitored by ^1^H and ^13^C NMR spectroscopy, and by TLC on Silufol UV-254 plates with acetone as eluent, visualized by iodine vapors.

### 3.2. Spectral Measurements

NMR spectra were registered on a Bruker DPX 400 spectrometer at working frequencies 400.13 MHz (^1^H NMR) and 100.61 MHz (^13^C) in DMSO-*d*_6_ or acetone-*d*_6_. Chemical shifts are given in parts per million (ppm) relative to the residual signals of the solvent (DMSO: H 2.49, C 39.5; acetone: H 2.04, C 29.8). Coupling constants are reported in hertz (Hz). Abbreviations: s, singlet; d, doublet; t, triplet; q, quartet; brs, broad singlet. UV spectra were taken on a Perkin UV-Vis Lambda 35 spectrometer for 10^−4^ M (products **3**, **6**) or 10^−3^ M (products **4**, **5**) acetonitrile solutions. The spectra of iodides have absorption maxima at 192–203 and 206–222 nm, whereas in the spectra of triiodides, longwave absorption bands at 289–298 and 359–361 nm were observed, typical for I_3_^−^ anion [33].

### 3.3. Synthesis

Reaction of 2-amino-1,3-benzothiazole (**1**) with α-iodomethyl ketones (**2a**–**f**) and diiodoacetone (**7**) (general procedure).

A mixture of 2-amino-1,3-benzothiazole **1** [0.20 g (1.3 mmol)] and an equimolar amount of α-iodomethyl ketones **2a** (0.24 g), **2b** (0.34 g), **2c** (0.43 g), **2d** (0.33 g), **2e** (0.38 g), **2f** (0.45 g) or 1,3-diiodoacetone **7** (0.31 g, 1.0 mmol) in 2 mL of dry acetone was stirred at room temperature for 6–9 h. The precipitated products **3a**–**f** were collected, washed with acetone and ether and dried under vacuum.

#### 3.3.1. 2-Amino-3-(2-oxopropyl)-1,3-benzothiazol-3-ium iodide (**3a**)

Yield 0.32 g (73%), light yellow powder, m. p. 182–184 °C. Anal. Calcd. for C_10_H_11_N_2_IOS: C, 35.94; H, 3.32; N, 8.38; I, 37.97; S, 9.60; Found: C, 36.18; H, 3.25; N, 8.41; I, 38.08; S, 9.53. ^1^H NMR (DMSO-*d*_6_, δ, ppm): 10.15 (br.s, 2H, NH_2_); 8.05 (d, 1H, H^7^, ^3^*J*_HH_ 7.2 Hz); 7.68 (d, 1H, H^4^, ^3^*J*_HH_ 7.3 Hz); 7.49 (d.d, 1H, H^5^, ^3^*J*_HH_ 7.3, 7.7 Hz); 7.39 (d.d, 1H, H^6^, ^3^*J*_HH_ 7.2, 7.7 Hz); 5.43 (s, 2H, CH_2_); 2.34 (s, 3H, Me). ^13^C NMR (DMSO-*d*_6_, δ, ppm: 199.96 (C=O); 169.02 (C^2^); 138.97 (C^9^); 128.32 (C^5^); 125.89 (C^6^); 124.27 (C^7^); 122.36 (C^8^); 113.81 (C^4^); 55.07 (CH_2_); 28.03 (Me). UV spectrum (MeCN), λ_max_, nm: 203, 222.

#### 3.3.2. 2-Amino-3-[2-(4-methylphenyl)-2-oxoethyl]-1,3-benzothiazol-3-ium iodide (**3b**)

Yield 0.32 g (60%), light yellow powder, m. p. 202–204 °C. Anal. Calcd. for C_16_H_15_N_2_IOS: C, 46.84; H, 3.68; N, 6.83; I, 30.93; S, 7.82; Found: C, 46.76; H, 3.55; N, 6.80; I, 31.05; S, 7.73. ^1^H NMR (DMSO-*d*_6_, δ, ppm): 10.21 (br.s, 2H, NH_2_); 8.08 (d, 1H, H^7^, ^3^*J*_HH_ 6.3 Hz); 7.03–8.05 (m, 2H, H*^o^*); 7.68 (d, 1H, H^4^, ^3^*J*_HH_ 6.3 Hz); 7.31–7.59 (m, 3H, H^5,6,*m*^); 6.08 (s, 2H, CH_2_); 2.44 (s, 3H, Me). ^13^C NMR (DMSO-*d*_6_, δ, ppm: 190.09 (C=O); 169.42 (C^2^); 145.62 (C*^p^*); 139.04 (C*^i^*); 131.82 (C^9^); 129.81 (C*^m^*); 129.17 (C*^o^*); 128.30 (C^8^); 125.83 (C^6^); 124.15 (C^5^), 122.33 (C^7^); 113.75 (C^4^); 52.48 (CH_2_); 21.80 (Me). UV spectrum (MeCN), λ_max_, nm: 203, 217.

#### 3.3.3. 2-Amino-3-[2-(1,1′-biphenyl)-4-yl-2-oxoethyl]-1,3-benzothiazol-3-ium iodide (**3c**)

Yield 0.33 g (54%), light yellow powder, m. p. 230–232 °C. Anal. Calcd. for C_21_H_17_N_2_IOS: C, 53.40; H, 3.63; N, 5.93; I, 26.87; S, 6.79; Found: C, 53.29; H, 3.57; N, 5.82; I, 26.97; S, 6.70. ^1^H NMR (DMSO-*d*_6_, δ, ppm): 10.27 (br.s, 2H, NH_2_); 8.20 (d, 2H, H*^o^*, ^3^*J*_HH_ 7.5 Hz); 8.10 (d, 1H, H^7^, ^3^*J*_HH_ 7.8 Hz); 7.98 (d, 2H, H*^m^*, ^3^*J*_HH_ 7.5 Hz); 7.81 (d, 2H, H^o′^, ^3^*J*_HH_ 7.3 Hz); 7.75 (d, 1H, H^4^, ^3^*J*_HH_ 7.6 Hz); 7.38–7.62 (m, 5H, H^5,6,*m*′,*p*^); 6.16 (s, 2H, CH_2_). ^13^C NMR (DMSO-*d*_6_, δ, ppm: 190.21 (C=O); 169.42 (C^2^); 146.16 (C*^p^*); 139.07 (C*^i^*^′^); 139.02 (C*^i^*); 133.11 (C^9^); 129.84 (C*^m^*); 129.61 (C*^o^*); 129.15 (C^5^); 128.31 (C*^p’^*); 127.52 (C*^m^*^′^); 127.38 (C*^o^*^′^); 125.85 (C^6^); 124.17 (C^8^); 122.35 (C^7^); 113.79 (C^4^); 52.66 (CH_2_). UV spectrum (MeCN), λ_max_, nm: 203, 221.

#### 3.3.4. 2-Amino-3-[2-(2-thienyl)-2-oxoethyl]-1,3-benzothiazol-3-ium iodide (**3d**)

Yield 0.36 g (70%), light yellow powder, m. p. 194–196 °C. Anal. Calcd. for C_13_H_11_N_2_IOS_2_: C, 38.81; H, 2.76; N, 6.96; I, 31.55; S, 15.94; Found: C, 38.73; H, 2.71; N, 6.89; I, 31.65; S, 15.89. ^1^H NMR (DMSO-*d*_6_, δ, ppm): 10.28 (br.s, 2H, NH_2_); 8.24–8.33 (m, 1H, H^3′^); 8.16–8.24 (m, 1H, H^5′^); 8.02–8.12 (m, 1H, H^7^); 7.64–7.75 (m, 1H, H^4^); 7.48–7.57 (m, 1H, H^5^); 7.37–7.48 (m, 2H, H^6,4’^); 6.04 (s, 2H, CH_2_). ^13^C NMR (DMSO-*d*_6_, δ, ppm: 183.33 (C=O); 169.15 (C^2^); 139.62 (C^8^); 138.48 (C^2′^); 136.45 (C^4′^); 135.39 (C^5′^); 129.15 (C^3′^); 127.92 (C^5^); 125.48 (C^6^); 123.74 (C^7^); 121.84 (C^9^); 113.24 (C^4^); 51.59 (CH_2_). UV spectrum (MeCN), λ_max_, nm: 202, 221.

#### 3.3.5. 2-Amino-3-[2-(5-chloro-2-thienyl)-2-oxoethyl]-1,3-benzothiazol-3-ium iodide (**3e**)

Yield 0.34 g (59%), light yellow powder, m. p. 210–213 °C. Anal. Calcd. for C_13_H_10_N_2_ClIOS_2_: C, 35.75; H, 2.31; N, 6.41; Cl, 8.12; I, 29.06; S, 14.68; Found: C, 35.68; H, 2.28; N, 6.37; Cl, 7.98; I, 29.17; S, 14.60. ^1^H NMR (DMSO-*d*_6_, δ, ppm): 10.31(br.s, 2H, NH_2_); 8.12–8.24 (m, 1H, H^3′^); 7.96–8.11 (m, 1H, H^7^); 7.61–7.82 (m, 1H, H^4^); 7.32–7.60 (m, 3H, H^5,6,4′^); 6.01 (s, 2H, CH_2_). ^13^C NMR (DMSO-*d*_6_, δ, ppm: 183.28 (C=O); 169.53 (C^2^); 139.41 (C^8^); 138.96 (C^2′^); 138.77 (C^5’^); 136.07 (C^4’^); 129.74 (C^3’^); 128.28 (C^5^); 125.86 (C^6^); 124.14 (C^7^); 122.22 (C^9^); 113.59 (C^4^); 51.70 (CH_2_). UV spectrum (MeCN), λ_max_, nm: 202, 222.

#### 3.3.6. 2-Amino-3-[2-(9*H*-carbazol-2-yl)-2-oxoethyl]-1,3-benzothiazol-3-ium iodide (**3f**)

Yield 0.32 g (50%), light yellow powder, m. p. 256–258 °C. Anal. Calcd. for C_21_H_16_IN_3_OS: C, 51.97; H, 3.32; N, 8.66; I, 26.15; S, 6.61; Found: C, 51.89; H, 3.28; N, 8.60; I, 26.25; S, 6.57. ^1^H NMR (DMSO-*d*_6_, δ, ppm): 10.36 (br.s, NH); 8.34–8.42 (m, 1H, H^7^); 8.23–8.33 (m, 2H, H^1′,4′^); 8.05–8.12 (m, 1H, H^5′^); 7.89–7.98 (m, 1H, H^3′^); 7.71–7.78 (m, 1H, H^4^); 7.58–7.66 (m, 1H, H^6^); 7.41–7.57 (m, 3H, 5,7’,8’); 7.21–7.29 (m, 11H, Ar); 6.26 (s, 2H, CH_2_). ^13^C NMR (DMSO-*d*_6_, δ, ppm): 190.38 (C=O); 169.46 (C^2^); 142.00; 139.15; 131.18; 128.29; 127.95; 127.54; 125.88; 125.81; 124.09; 122.38; 121.92; 121.85; 120.72; 119.79; 119.19; 113.84; 112.45; 112.00 (Ar); 52.66 (CH_2_). UV spectrum (MeCN), λ_max_, nm: 203, 222.

##### Reaction of Iodides (**3a**–**f**) and (**6a**–**f**) with Elemental Iodine (General Procedure)

A mixture of equimolar amounts (1.00 mmol) of iodides **3a**–**f** or **6a**–**f** and elemental iodine in 5 mL of acetone was stirred for 2–3 h at room temperature. After completion, the products **4a**–**f** or **5a**–**f** were precipitated by the addition of 35 mL of hexane, washed with cooled ether and dried under vacuum.

#### 3.3.7. 2-Amino-3-[2-(2-thienyl)-2-oxoethyl]-1,3-benzothiazol-3-ium triiodide (**4d**)

Yield 0.59 g (91%), dark red powder, m. p. 124–126 °C. Anal. Calcd. for C_13_H_11_N_2_I_3_OS_2_: C, 23.80; H, 1.69; N, 4.27; I, 58.03; S, 9.77; Found: C, 23.69; H, 1.60; N, 4.20; I, 58.15; S, 9.68. ^1^H NMR (DMSO-*d*_6_), δ, ppm: 10.27 (br.s, 2H, NH_2_); 8.25 (d, 1H, H^3′^, ^3^*J*_HH_ 3.4 Hz); 8.19 (d, 1H, H^5′^, ^3^*J*_HH_ 5.0 Hz); 8.02 (d, 1H, H*^7^*, ^3^*J*_HH_ 7.6 Hz); 7.68 (d, 1H, H^4^, ^3^*J*_HH_ 8.2 Hz); 7.52 (d.d, 1H, H^5^, *J*_HH_ 7.0, 8.2 Hz); 7.39–7.48 (m, 2H, H^6,4′^); 6.00 (s, 2H, CH_2_). ^13^C NMR (DMSO-*d*_6_), δ, ppm: 183.85 (C=O); 169.74 (C^2^); 140.19 (C^8^); 139.07 (C^2′^); 136.97 (C^4′^); 135.87 (C^5′^); 129.70 (C^3′^); 128.50 (C^5^); 126.08 (C^6^); 124.23 (C^7^); 122.36 (C^9^); 113.80 (C^4^); 52.04 (CH_2_). UV spectrum (MeCN), λ_max_, nm: 294, 361.

#### 3.3.8. 2-Amino-3-[2-(5-chloro-2-thienyl)-2-oxoethyl]-1,3-benzothiazol-3-ium triiodide (**4e**)

Yield 0.62 g (90%), dark red powder, m. p. 199–201 °C. Anal. Calcd. for C_13_H_10_N_2_ClI_3_OS_2_: C, 22.61; H, 1.46; N, 4.07; Cl, 5.13; I, 55.13; S, 9.29; Found: C, 22.56; H, 1.40; N, 3.97; Cl, 4.97; I 55.23; S, 9.20. ^1^H NMR (acetone-*d*_6_), δ, ppm: 9.78 (br.s, 2H, NH_2_); 8.18 (d, 1H, H^3′^,^3^*J*_HH_ 3.8 Hz); 8.07 (d, 1H, H*^7^*, ^3^*J*_HH_ 8.3 Hz); 7.80 (d, 1H, H^4^, ^3^*J*_HH_ 8.3 Hz); 7.62 (d.d, 1H, H^5^, ^3^*J*_HH_ 7.6, 8.3 Hz); 7.54 (d.d, 1H, H^6^, ^3^*J*_HH_ 7.6, 8.3 Hz); 7.36 (d, 1H, H^4′^, ^3^*J*_HH_ 3.8 Hz); 6.23 (s, 2H, CH_2_). ^13^C NMR, acetone-*d*_6_, δ, ppm: 182.49 (C=O); 170.98 (C^2^); 141.10 (C^9^); 139.56 (C^2′^); 139.40 (C^5′^); 135.79 (C^4′^); 129.77 (C^3′^), 129.18 (C^5^); 127.02 (C^6^); 124.34 (C^7^); 122.75 (C^8^); 114.23 (C^4^); 52.24 (CH_2_). UV spectrum (MeCN), λ_max_, nm: 291, 361.

#### 3.3.9. 2-Methyl-1*H*-imidazo[2,1-b][1,3]benzothiazol-4-ium triiodide (**5a**)

Yield 0.56 g (93%), dark red powder, m. p. 154–156 °C. Anal. Calcd. for C_10_H_9_N_2_I_3_S. C, 21.07; H, 1.59; N, 4.91; I, 66.80; S, 5.62; Found: C, 20.91; H, 1.51; N, 4.87; I, 66.90; S, 5.58. ^1^H NMR (DMSO-*d*_6_, δ, ppm): 8.46 (s, 1H, CH=); 8.17–8.27 (m, 2H, H^4,7^); 7.70 (d.d, 1H, H^5^, ^3^*J*_HH_ 7.7, 8.6 Hz); 7.61 (d.d, 1H, H^6^, ^3^*J*_HH_ 7.5, 7.7 Hz); 2.46 (s, 3H, Me). ^13^C NMR (DMSO-*d*_6_, δ, ppm): 145.92 (C^10^); 135.77(C^2^); 131.67 (C^11^); 129.89 (C^12^); 128.29 (C^8^); 127.62 (C^6^); 126.04 (C^7^); 115.37 (C^3^); 112.32 (C^5^); 11.87 (CH_3_). UV spectrum (MeCN), λ_max_, nm: 289, 361.

#### 3.3.10. 6-(4-Methylphenyl)-7*H*-imidazo[2,1-b][1,3]benzothiazol-4-ium triiodide (**5b**)

Yield 0.39 g (89%), dark red powder, m. p. 198–200 °C. Anal. Calcd. for C_16_H_13_N_2_I_3_S. C, 29.74; H, 2.03; N, 4.34; I, 58.93; S, 4.96; Found: C, 29.65; H, 1.96; N, 4.28; I, 59.05; S, 4.88. ^1^H NMR (acetone-*d*_6_, δ, ppm): 9.09 (s, 1H, CH=); 8.32–8.41 (m, 2H, H^5,8^); 7.89 (d.d, 1H, H^6^, ^3^*J*_HH_ 7.7, 7.9 Hz); 7.75–7.82 (m, 3H, H^7,*o*^); 7.46 (d, 1H, H*^m^*, ^3^*J*_HH_ 7.9 Hz); 2.44 (s, 3H, Me). ^13^C NMR (acetone-*d*_6_, δ, ppm): 144.10 (C^10^); 140.90 (C*^p^*); 135.67 (C^11^); 132.19 (C^2^); 130.75 (C*^m^*); 130.31 (C*^i^*); 129.63 (C^12^); 128.56 (C^8^); 128.17 (C^6^); 125.61 (C^7^); 125.15 (C*^o^*); 115.15 (CH=); 110.38 (C^5^); 22.55 (CH_3_). UV spectrum (MeCN), λ_max_, nm: 295, 361.

#### 3.3.11. 2-(1,1′-Biphenyl)-4-yl-)-1*H*-imidazo[2,1-b][1,3]benzothiazol-4-ium triiodide (**5c**)

Yield 0.60 g (86%), dark red powder, m. p. 170–171 °C. Anal. Calcd. for C_21_H_15_N_2_I_3_S. C, 35.62; H, 2.14; N, 3.96; I, 53.76; S, 4.53; Found: C, 35.53; H, 2.08; N, 3.90; I, 53.86; S, 4.47. ^1^H NMR (acetone-*d*_6_, δ, ppm): 9.00 (s, 1H, CH=); 8.20–8.27 (m, 2H, H^5,8^); 8.03 (d, 2H, H*^o^*^’^*,* ^3^*J*_HH_ 6.4 Hz); 7.86 (d, 2H, H*^o^,* ^3^*J*_HH_ 7.9 Hz); 7.70–7.81 (m, 3H, H*^m^*^,*p*′^); 7.67 (d.d, 1H, H^6^, ^3^*J*_HH_ 7.6, 7.9 Hz); 7.47–7.54 (m, 2H, H*^m^*^′^); 7.42 (d.d, 1H, H^7^, ^3^*J*_HH_ 7.6, 7.9 Hz). ^13^C NMR (acetone-*d*_6_, δ, ppm): 147.36 (C^10^); 145.16 (C*^p^*); 139.76 (C^11^); 139.35 (C*^i^*^′^); 132.21 (C*^i^*); 131.82 (C^2^); 129.65 (C^8^); 129.37 (C*^m^*); 129.22 (C^6^); 127.74 (C*^m^*^′^); 127.23 (C^7^); 127.10 (C*^o^*); 126.64 (C*^p^*^′^); 125.75 (C*^o^*^′^); 125.55 (C^12^); 113.76 (C^3^); 109.51 (C^5^). UV spectrum (MeCN), λ_max_, nm: 298, 361.

#### 3.3.12. 2-(2-Thienyl)-1*H*-imidazo[2,1-b][1,3]benzothiazol-4-ium triiodide (**5d**)

Yield 0.55 g (88%), dark red powder, m. p. 196–198 °C. Anal. Calcd. for C_13_H_9_N_2_I_3_S_2_. C, 24.47; H, 1.42; N, 4.39; I, 59.67; S, 10.05; Found: C, 24.39; H, 1.36; N, 4.32; I, 59.78; S, 9.97. ^1^H NMR (acetone-*d*_6_, δ, ppm): 8.96 (s, 1H, CH=); 8.29–8.37 (m, 2H, H^5,8^); 7.85 (d.d, 1H, H^6^, ^3^*J*_HH_ 7.4, 7.6 Hz); 7.69–7.78 (m, 3H, H^7,3′,5′^); 7.27 (d.d, 1H, H^4′^, ^3^*J*_HH_ 4.3, 5.3 Hz). ^13^C NMR (acetone-*d*_6_, δ, ppm): 148.23 (C^10^); 134.90 (C^2^); 132.39 (C^11^); 130.36 (C^2′^); 129.35 (C^3′^); 129.16 (C^5′^); 129.07 (C^8^); 128.69 (C^6^); 128.21 (C^7^); 126.28 (C^4′^); 125.21 (C^12^); 115.92 (C^3^); 111.10 (C^5^). UV spectrum (MeCN), λ_max_, nm: 289, 361.

#### 3.3.13. 2-(2-(5-Chloro-2-thienyl))-1*H*-imidazo[2,1-b][1,3]benzothiazol-4-ium triiodide (**5e**)

Yield 0.55 g (84%), dark red powder, m. p. 191–193 °C. Anal. Calcd. for C_13_H_8_N_2_ClI_3_S_2_. C, 23.22; H, 1.20; N, 4.16; I, 56.61; S, 9.54; Found: C, 23.14; H, 1.16; N, 4.07; I, 56.72; S, 9.48. ^1^H NMR (DMSO-*d*_6_, δ, ppm): 8.61 (s, 1H, CH=); 7.94–8.03 (m, 2H, H^5,8^); 7.54 (d.d, 1H, H^6^, ^3^*J*_HH_ 7.9, 8.1 Hz); 7.42 (d.d, 1H, H^7^, ^3^*J*_HH_ 7.0, 7.9 Hz); 7.25 (d, 1H, H^3′^, ^3^*J*_HH_ 3.5 Hz); 7.08 (d, 1H, H^4′^, ^3^*J*_HH_ 3.5 Hz). ^13^C NMR (DMSO-*d*_6_, δ, ppm): 147.39 (C^10^); 140.41 (C^2^); 136.57 (C^11^); 131.73 (C^2′^); 129.40 (C^5′^); 127.99 (C^12^); 127.06 (C^3′^); 126.76 (C^8^); 125.71 (C^6^); 125.23 (C^4′^); 122.24 (C^7^); 113.72 (C^3^); 108.81 (C^5^). UV spectrum (MeCN), λ_max_, nm: 289, 361.

#### 3.3.14. 2-(2-(9*H*-Carbazol-2-yl)-)-1*H*-imidazo[2,1-b][1,3]benzothiazol-4-ium triiodide (**5f**)

Yield 0.58 g (83%), dark red powder, m. p. 258–260 °C. Anal. Calcd. for C_21_H_14_I_3_N_3_S: C, 34.98; H, 1.96; N, 5.83; I, 52.79; S, 4.45. Found: C, 34.89; H, 1.90; N, 5.77; I, 52.91; S, 4.39. ^1^H NMR (DMSO-*d*_6_, δ, ppm): 11.50 (br.s, NH); 9.12 (s, 1H, H^3^); 8.09–8.28 (m, 4H, H^5,4′,5′,8^); 7.94 (s, 1H, H^1′^); 7.71 (d.d, 1H, H^6^*,* ^3^*J*_HH_ 7.6, 8.4 Hz); 7.50–7.65 (m, 3H, H^3′,7,8′^); 7.41 (d.d, 1H, H^7′^, ^3^*J*_HH_ 7.4, 7.6 Hz); 7.18 (d.d, 1H, H^6′^*,* ^3^*J*_HH_ 7.4, 7.6 Hz). ^13^C NMR (DMSO-*d*_6_, δ, ppm): 155.17 (C^10^); 147.10; 141.18; 140.85; 140.15; 131.67; 129.53; 128.08; 127.25; 126.61; 125.91; 123.47; 122.32; 121.47; 120.83; 119.43; 116.29; 114.95; 111.48; 110.51; 107.79 (Ar). UV spectrum (MeCN), λ_max_, nm: 292, 359.

##### Cyclization of Iodides (**3a**–**f**)

(a) One mmol of iodides **3a**–**f** [**3a** 0.33 g, **3b** 0.42 g, **3c** 0.47 g, **3d** 0.40 g, **3e** 0.44g, **3f** 0.49 g] was placed in an ampoule with DMSO and kept for 1–24 h at room temperature, with monitoring by ^1^H NMR after 1, 12, and 24 h. Then, the reaction mixture was added dropwise to 50 mL of diethyl ether at vigorous stirring. The formed precipitate was filtered off, washed with ether and dried under vacuum.

(b) A suspension of 1 mmol of iodides **3a**–**f** in MeOH was stirred for 7 h at 40 °C, with monitoring by ^1^H NMR in 1, 3.5, and 7 h. Then, the reaction mixture was added dropwise to 40 mL of diethyl ether at vigorous stirring. The formed precipitate was filtered off, washed with ether and dried under vacuum.

#### 3.3.15. 2-Methyl-1*H*-imidazo[2,1-b][1,3]benzothiazol-4-ium iodide (**6a**)

Yield 0.28 g (86%), light yellow powder, m. p. 138–140 °C. Anal. Calcd. for C_10_H_9_N_2_IS. C, 37.99; H, 2.87; N, 8.86; I, 40.14; S, 10.14; Found: C, 37.88; H, 2.84; N, 8.79; I, 40.24; S, 10.03. ^1^H NMR (DMSO-*d*_6_, δ, ppm): 8.44 (s, 1H, CH=); 8.17–8.27 (m, 2H, H^4,7^); 7.69 (d.d, 1H, H^5^, ^3^*J*_HH_ 7.1, 8.1 Hz); 7.59 (d.d, 1H, H^6^, ^3^*J*_HH_ 8.1, 8.3 Hz); 2.44 (s, 3H, Me). ^13^C NMR (DMSO-*d*_6_, δ, ppm): 145.90 (C^10^); 136.41 (C^11^); 131.71 (C^2^); 129.81 (C^12^); 128.15 (C^8^); 127.38 (C^6^); 126.01 (C^7^); 115.21 (C^3^); 112.15 (C^5^); 12.05 (CH_3_). UV spectrum (MeCN), λ_max_, nm: 193, 210.

#### 3.3.16. 2-(4-Methylphenyl)-1*H*-imidazo[2,1-b][1,3]benzothiazol-4-ium iodide (**6b**)

Yield 0.25 g (63%), light yellow powder, m. p. 219–221 °C. Anal. Calcd. for C_16_H_13_N_2_IS. C, 48.99; H, 3.34; N, 7.14; I, 32.35; S, 8.17; Found: C, 48.93; H, 3.30; N, 7.08; I, 32.44; S, 8.10. ^1^H NMR (DMSO-*d*_6_, δ, ppm): 8.90 (s, 1H, CH=); 8.13 (d, 1H, H^5^, ^3^*J*_HH_ 7.9 Hz); 8.08 (d, 1H, H^8^, ^3^*J*_HH_ 8.2 Hz); 7.72 (d, 2H, H*^o^*, ^3^*J*_HH_ 7.8 Hz); 7.64 (d.d, 1H, H^6^, ^3^*J*_HH_ 7.9, 7.8 Hz); 7.51 (d.d, 1H, H^7^, ^3^*J*_HH_ 7.8, 8.2 Hz); 7.28 (d, 2H, H*^m^*, ^3^*J*_HH_ 7.8 Hz); 2.33 (s, 3H, Me). ^13^C NMR (DMSO-*d*_6_, δ, ppm): 147.28 (C^10^); 143.60 (C*^p^*); 138.11 (C^11^); 132.06 (C^2^); 130.07 (C*^m^*); 129.68 (C*^i^*); 128.90 (C^12^); 127.68 (C^8^); 126.48 (C^6^); 125.78 (C^7^); 125.30 (C*^o^*); 114.39 (CH=); 109.43 (C^5^); 22.01 (CH_3_). UV spectrum (MeCN), λ_max_, nm: 192, 206.

#### 3.3.17. 2-(1,1’-Biphenyl)-4-yl-)-1*H*-imidazo[2,1-b][1,3]benzothiazol-4-ium iodide (**6c**)

Yield 0.30 g (67%), light yellow powder, m. p. 188–190 °C. Anal. Calcd. for C_21_H_15_N_2_IS. C, 55.52; H, 3.33; N, 6.16; I, 27.93; S, 7.06; Found: C, 55.46; H, 3.28; N, 6.10; I, 28.02; S, 7.01. ^1^H NMR (DMSO-*d*_6_, δ, ppm): 8.76 (s, 1H, CH=); 7.94–8.00 (m, 2H, H^5,8^); 7.87–7.91 (m, 2H, H*^o^*); 7.67–7.71 (m, 2H, H*^m^*); 7.63–7.66 (m, 2H, H*^o^*^’^);7.53 (d.d, 1H, H^6^, ^3^*J*_HH_ 7.9, 8.1 Hz); 7.38–7.45 (m, 3H, H*^m^*^′*p*′^); 7.33 (d.d, 1H, H^7^, ^3^*J*_HH_ 7.3, 8.1 Hz). ^13^C NMR (DMSO-*d*_6_, δ, ppm): 147.58 (C^10^); 145.13 (C*^p^*); 140.01 (C^11^); 139.68 (C*^i^*^’^); 132.26 (C*^i^*); 132.07 (C^2^); 129.65 (C^8^); 129.47 (C*^m^*); 128.01 (C^6^); 127.50 (C*^m^*^’^); 127.37 (C^7^); 126.92 (C*^o^*); 126.04 (C*^p^*^′^); 125.81 (C*^o^*^′^); 125.50 (C^12^); 114.06 (C^3^); 109.83 (C^5^). UV spectrum (MeCN), λ_max_, nm: 192, 206.

#### 3.3.18. 2-(2-Thienyl)-1*H*-imidazo[2,1-b][1,3]benzothiazol-4-ium iodide (**6d**)

Yield 0.24 g (64%), light yellow powder, m. p. 206–208 °C. Anal. Calcd. for C_13_H_9_N_2_IS_2_. C, 40.63; H, 2.36; N, 7.29; I, 33.02; S, 16.69; Found: C, 40.57; H, 2.30; N, 7.21; I, 33.13; S, 16.61. ^1^H NMR (DMSO-*d*_6_, δ, ppm): 8.61 (s, 1H, CH=); 7.95–8.01 (m, 2H, H^5,8^); 7.54 (d.d, 1H, H^6^, ^3^*J*_HH_ 7.3, 8.4 Hz); 7.38–7.46 (m, 3H, H^7,3′,5′^); 7.10 (d.d, 1H, H^4′^, ^3^*J*_HH_ 3.8, 4.9 Hz). ^13^C NMR (DMSO-*d*_6_, δ, ppm): 146.99 (C^10^); 141.13 (C^2^); 137.08 (C^11^); 131.71 (C^2′^); 129.23 (C^3′^); 128.08 (C^5′^); 126.88 (C^8^); 125.44 (C^12^); 125.08 (C^6^); 124.88 (C^7^); 122.88 (C^4′^); 113.56 (C^5^); 108.39 (C^3^). UV spectrum (MeCN), λ_max_, nm: 192, 206.

#### 3.3.19. 2-(2-(5-Chloro-2-thienyl))-1*H*-imidazo[2,1-b][1,3]benzothiazol-4-ium iodide (**6e**)

Yield 0.25 g (62%), light yellow powder, m. p. 205–207 °C. Anal. Calcd. for C_13_H_8_N_2_ClIS_2_. C, 37.29; H, 1.92; N, 6.69; I, 30.31; S, 15.31; Found: C, 37.23; H, 1.88; N, 6.61; I, 30.71; S, 15.26. ^1^H NMR (acetone-*d*_6_, δ, ppm): 8.45 (s, 1H, CH=); 7.91–7.99 (m, 2H, H^5,8^); 7.57 (d.d, 1H, H^6^, ^3^*J*_HH_ 7.5, 7.8 Hz); 7.46 (d.d, 1H, H^7^, ^3^*J*_HH_ 7.5, 8.1 Hz); 7.27 (d, 1H, H^3′^, ^3^*J*_HH_ 3.6 Hz); 6.99 (d, 1H, H^4′^, ^3^*J*_HH_ 3.6 Hz). ^13^C NMR (acetone-*d*_6_, δ, ppm): 147.50 (C^10^); 140.51 (C^2^); 136.30 (C^11^); 131.04 (C^2′^); 129.76 (C^5′^); 127.73 (C^12^); 127.32 (C^3′^); 126.89 (C^8^); 125.61 (C^6^); 124.83 (C^4′^); 122.20 (C^7^); 113.47 (C^3^); 107.97 (C^5^). UV spectrum (MeCN), λ_max_, nm: 192, 206.

#### 3.3.20. 2-(2-(9*H*-Carbazol-2-yl)-)-1*H*-imidazo[2,1-b][1,3]benzothiazol-4-ium iodide (**6f**)

Yield 0.26 g (58%), light yellow powder, m. p. 248–250 °C. Anal. Calcd. for C_21_H_14_IN_3_S: C, 59.97; H, 3.02; N, 8.99; I, 27.16; S, 6.86; Found: C, 59.84; H, 2.78; N, 8.64; I, 27.45; S, 6.80. ^1^H NMR (DMSO-*d*_6_, δ, ppm): 11.43 (br.s, NH); 8.99 (s, 1H, H^3^); 8.19 (d, 1H, H^8^, ^3^*J*_HH_ 7.8 Hz); 8.07–8.16 (m, 3H, H^5,4′,3′^); 8.00 (s, 1H, H^1′^); 7.62–7.71 (m, 2H, H^7,8′^); 7.47–7.54 (m, 2H, H^5′,6^); 7.35–7.43 (m, 1H, H^7′^); 7.13–7.19 (m, 1H, H^6′^). ^13^C NMR (DMSO-*d*_6_, δ, ppm): 169.25 (C^10^); 146.82; 140.37; 140.09; 138.47; 131.68; 129.21; 129.07; 127.06; 125.76; 125.21; 122.28; 120.68; 120.21; 118.82; 115.96; 113.80; 111.01; 109.41; 107.16; 106.03 (Ar). UV spectrum (MeCN), λ_max_, nm: 203, 211.

### 3.4. Computational Details

All calculations were performed with full geometry optimization using the B3LYP/DGDZVP method as implemented in Gaussian 09 [34].

## 4. Conclusions

Based on the reaction of 2-amino-1,3-benzothiazole with α-iodoketones in the absence of bases and catalysts, both linear and cyclic iodide and triiodide salts with 2-amino-1,3-benzothiazol-3-ium and 6-R-7*H*-imidazo[2,1-*b*][1,3]benzothiazol-4-ium cations were synthesized. The experimentally observed preference for the endo *N*-alkylation is explained theoretically.

## Data Availability

Not applicable.

## Supplementary Materials

The following supporting information can be downloaded at: https://www.mdpi.com/article/10.3390/molecules28052093/s1. Copies of the ^1^H and ^13^C NMR spectra for all synthesized compounds can be found in Supplementary Materials.

## Sample Availability

Samples of the compounds are available from the authors.
