# Supplementary material for: 2-Amino-1,3-benzothiazole: Endo N-Alkylation with α-Iodo Methyl Ketones Followed by Cyclization
Source: Molecules. 2023 Feb 23;28(5):2093. doi: 10.3390/molecules28052093 (PMC10004639; doi:10.3390/molecules28052093)
Supplement: Supplementary file 1 [file molecules-28-02093-s001.zip › molecules-2208020-supplementary.pdf]

Supporting information

2-Amino-1,3-Benzothiazole: Endo *N*-Alkylation with *α*-Iodo Methyl Ketones Followed by Cyclization

Ivan A. Dorofeev, Larisa V. Zhilitskaya, Nina O. Yarosh and Bagrat A. Shainyan *

A. E. Favorsky Irkutsk Institute of Chemistry, Siberian Division of the Russian Academy of Sciences, 1 Favorsky Street, 664033 Irkutsk, Russia
* Correspondence: bagrat@irioch.irk.ru

Figure S1. ^{1}H NMR spectrum of compound (3a).

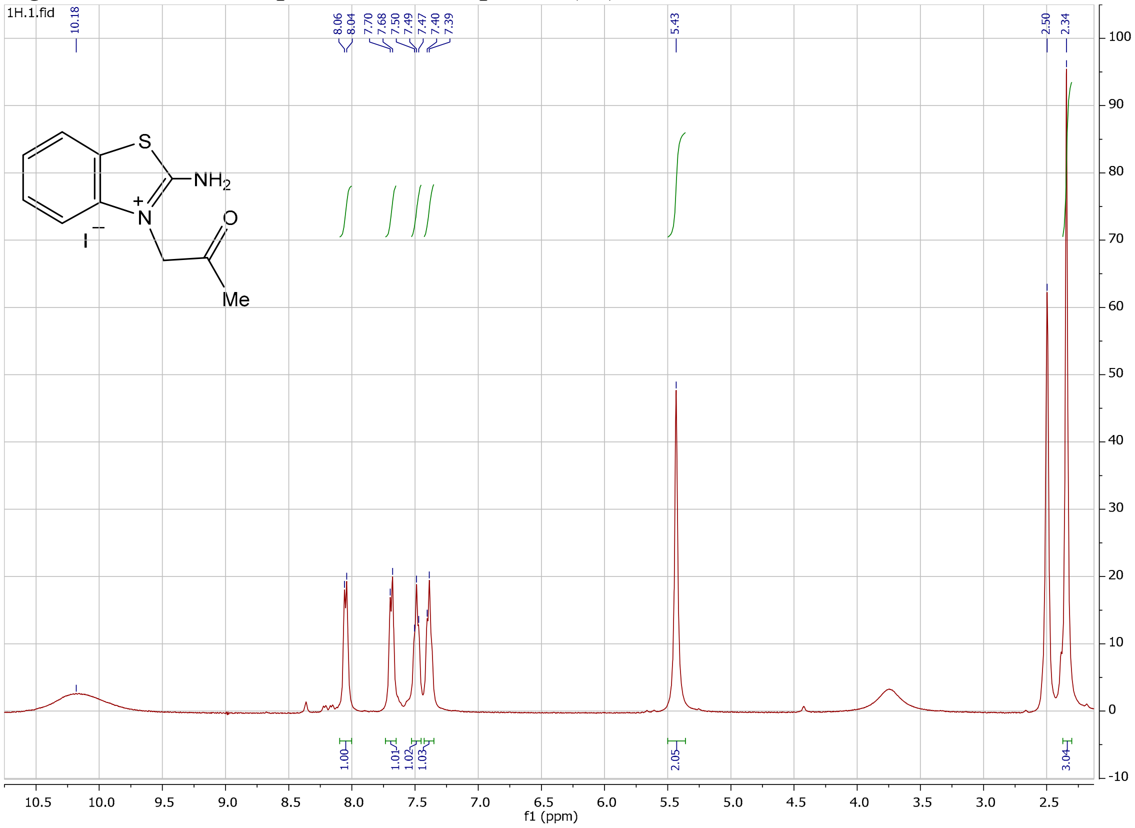

Figure S2. ^{13}C NMR spectrum of compound (3a).

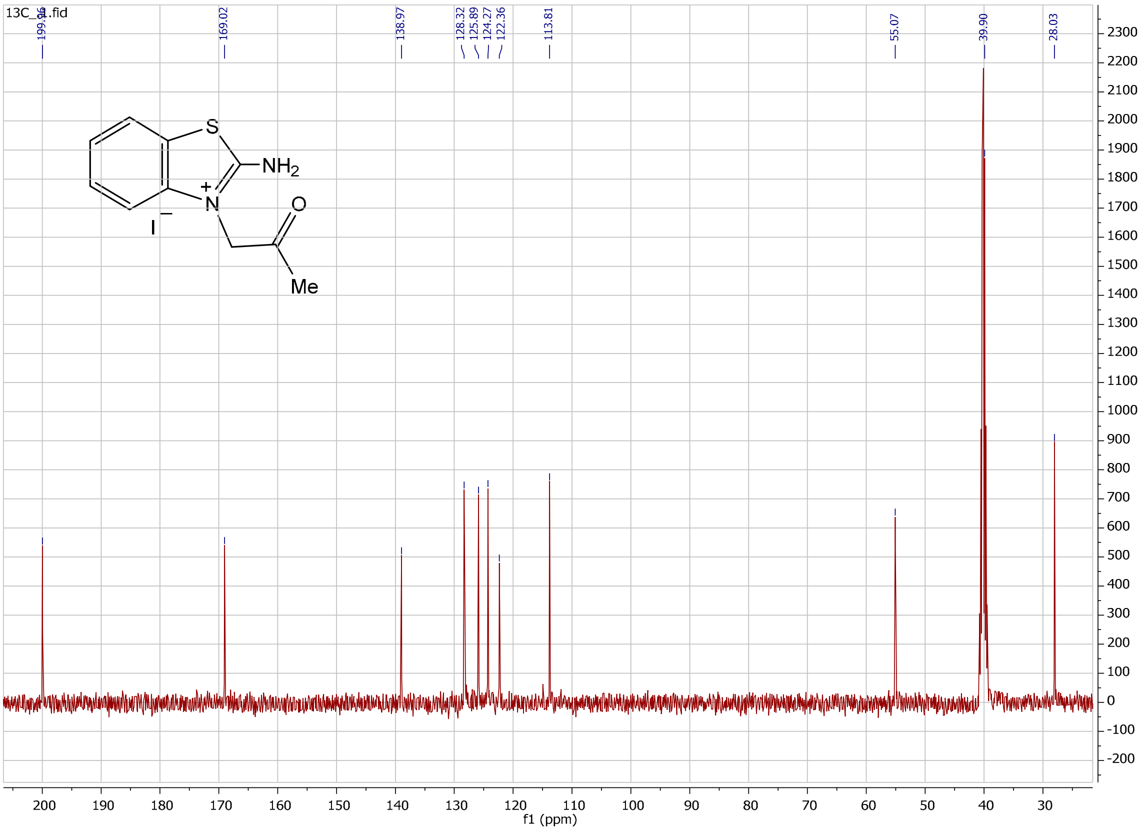

Figure S3. ^{1}H NMR spectrum of compound (3b).

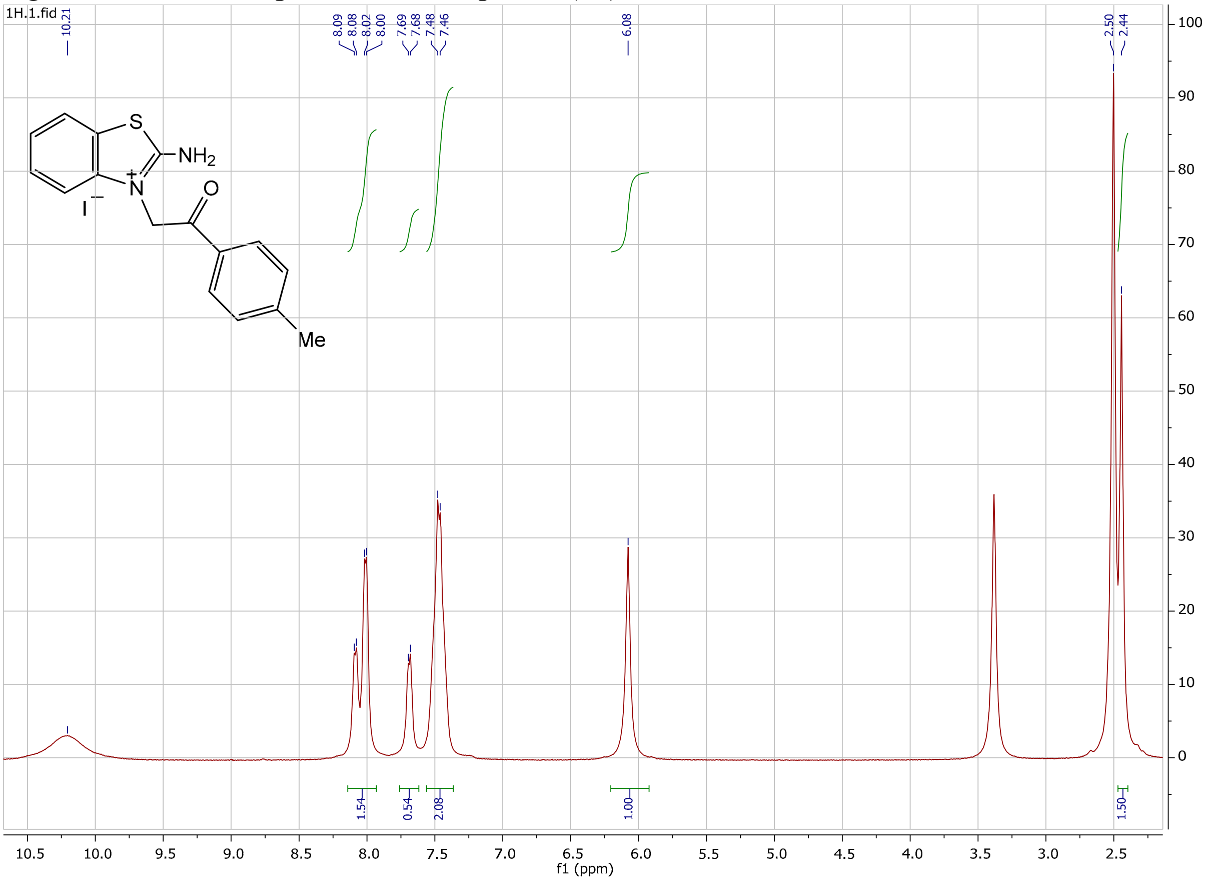

Figure S4. ^{13}C NMR spectrum of compound (3b).

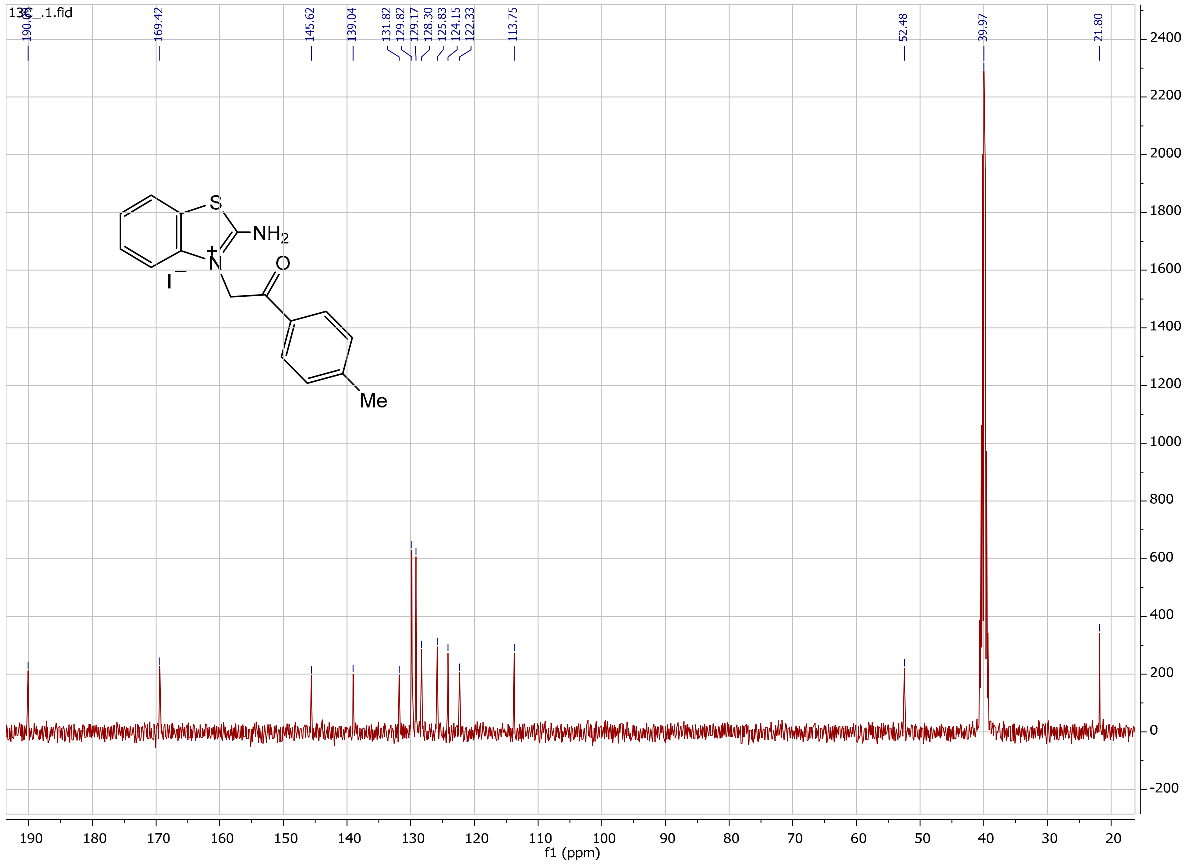

Figure S5. ^{1}H NMR spectrum of compound (3c).

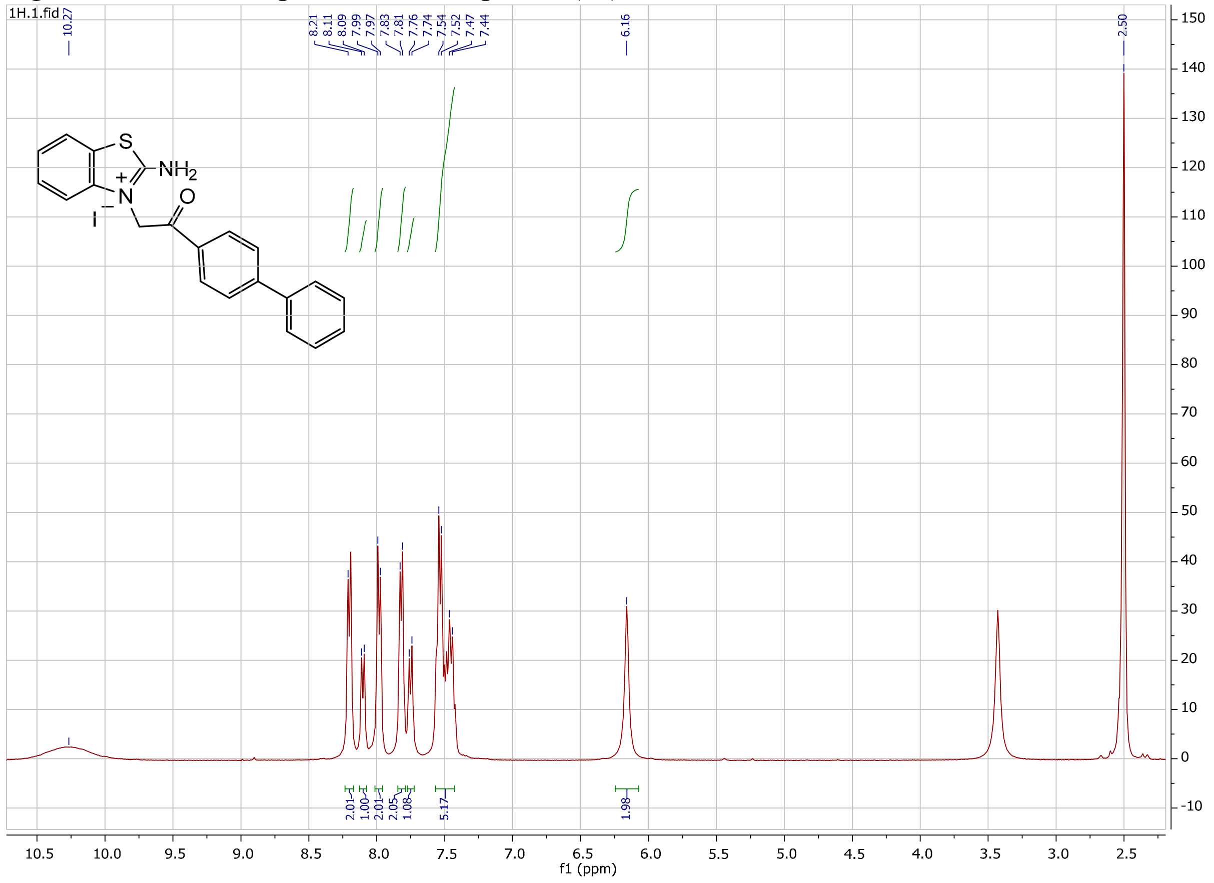

Figure S6. ^{13}C NMR spectrum of compound (3c).

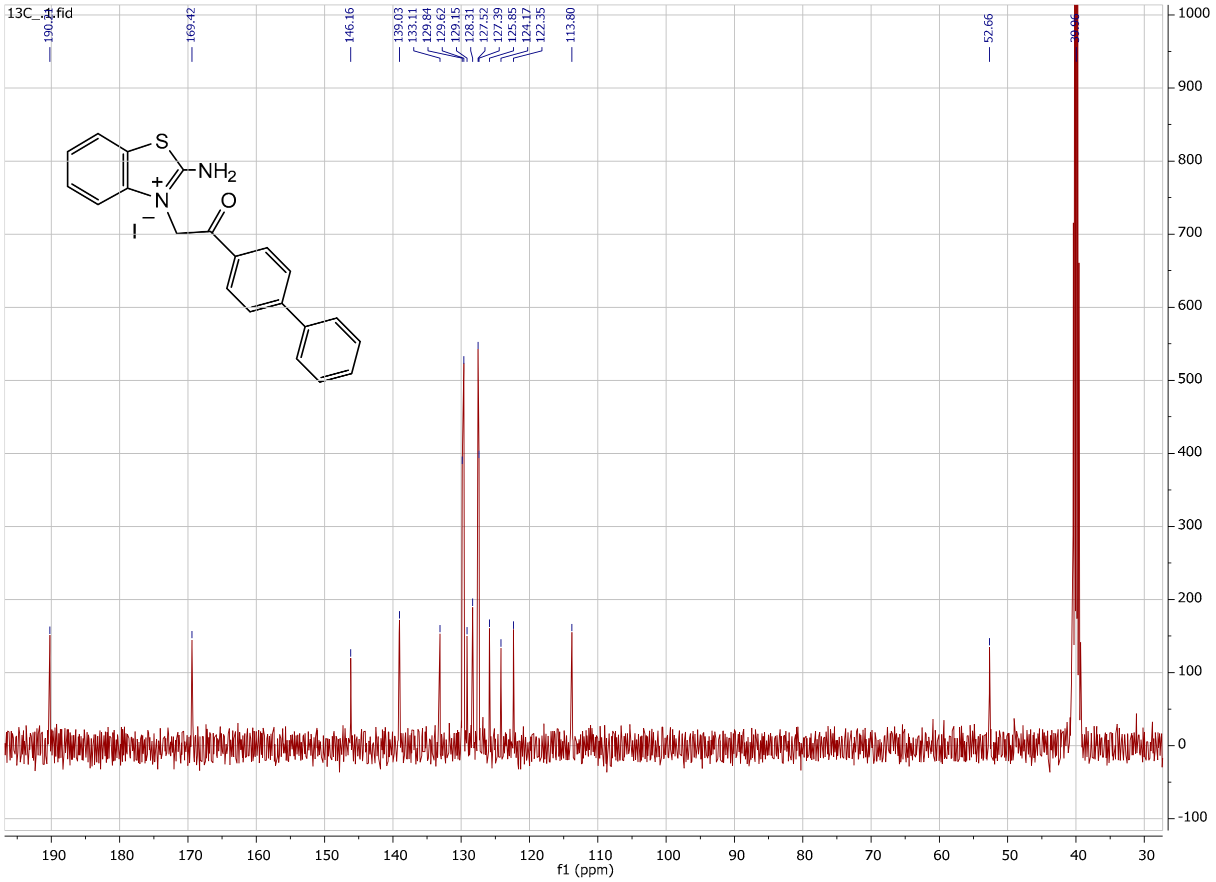

Figure S7. ^{1}H NMR spectrum of compound (3d).

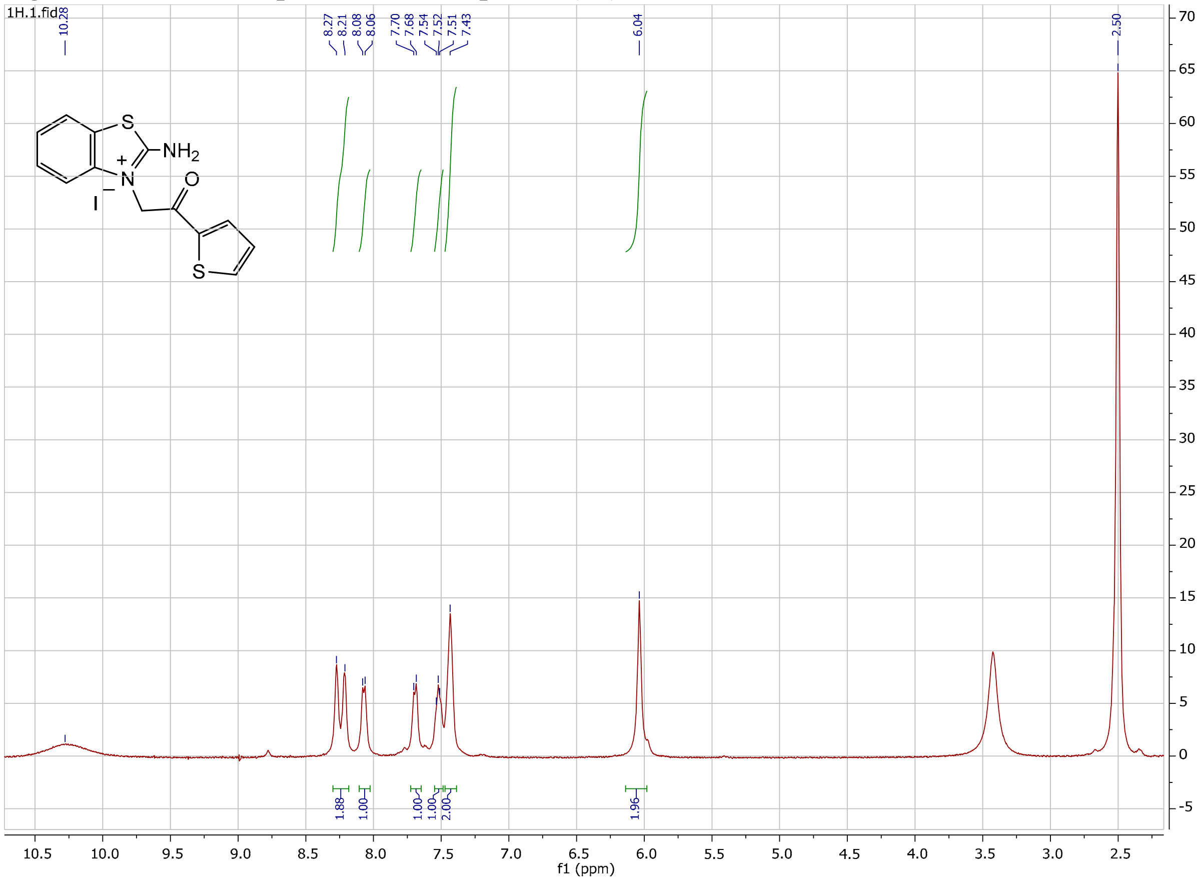

Figure S8. ^{13}C NMR spectrum of compound (3d).

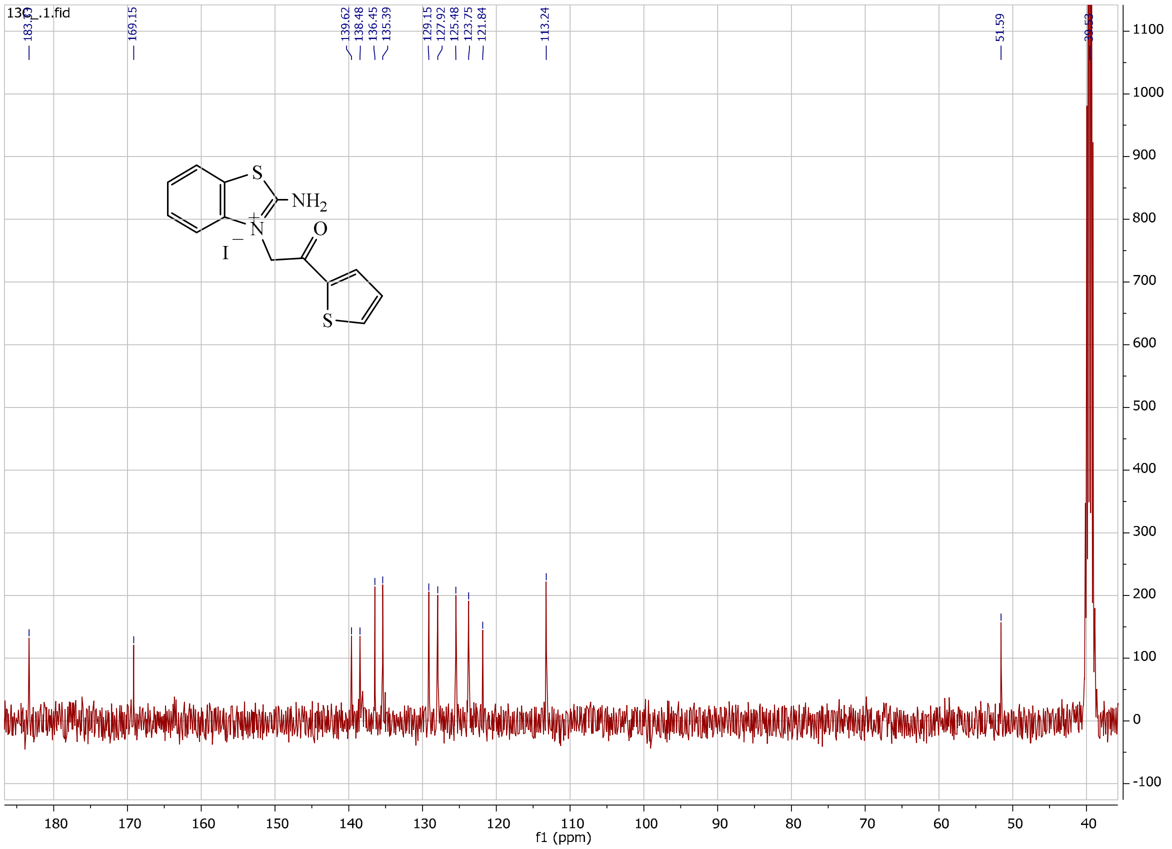

Figure S9. ^{1}H NMR spectrum of compound (3e).

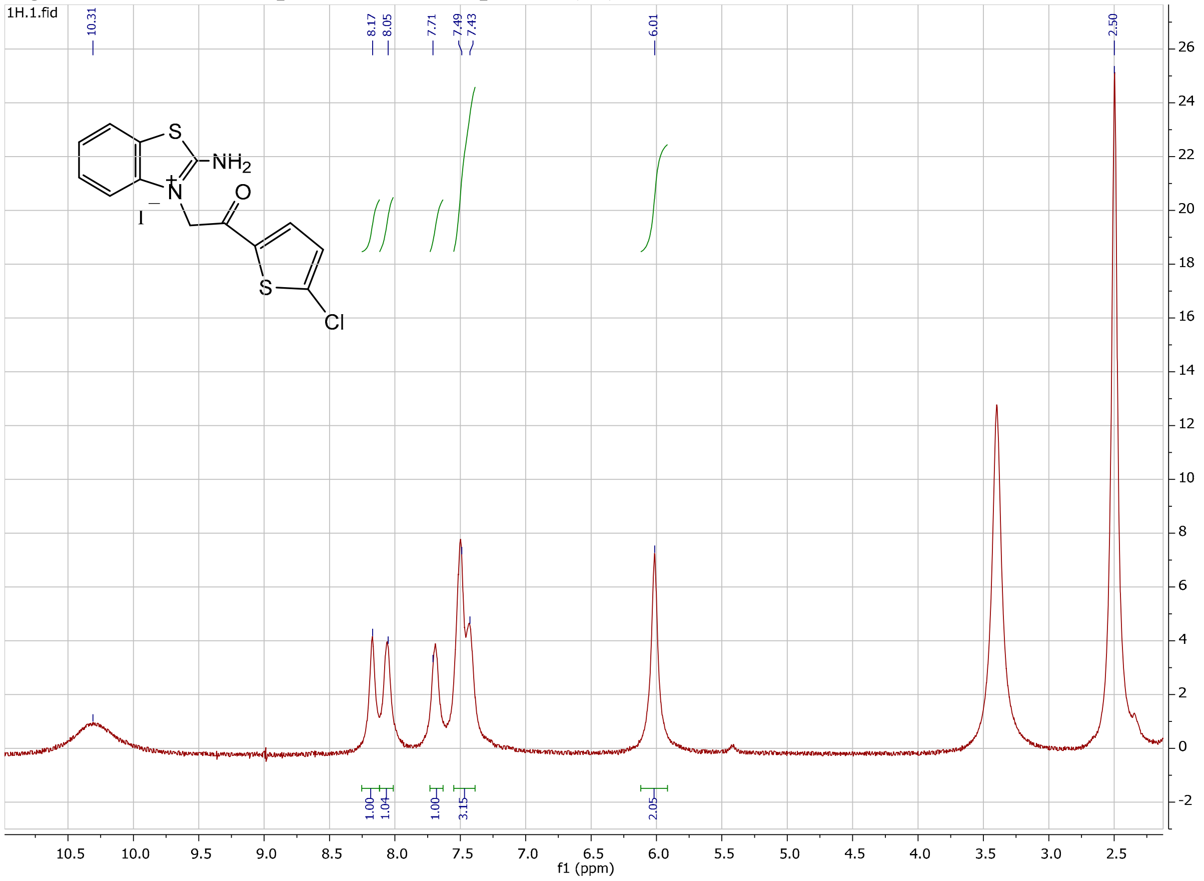

Figure S10. ^{13}C NMR spectrum of compound (3e).

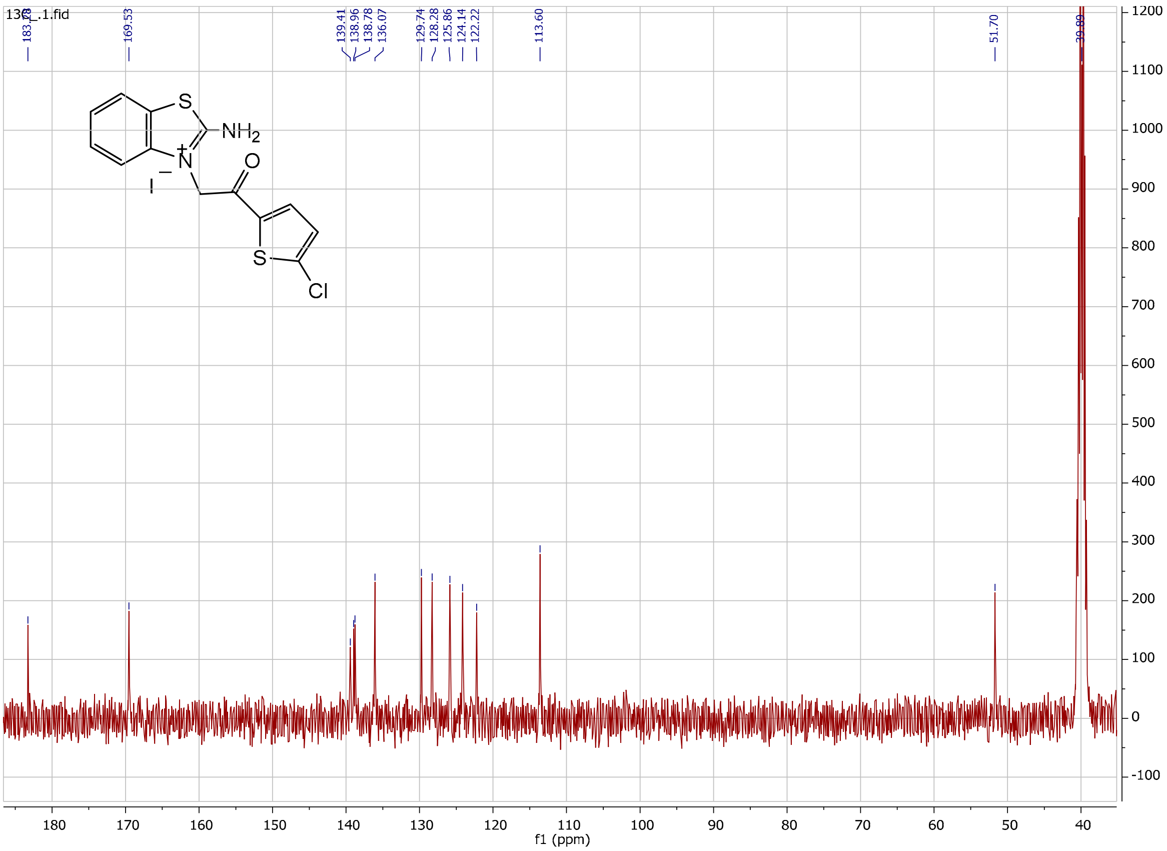

Figure S11. ^{1}H NMR spectrum of compound (3f).

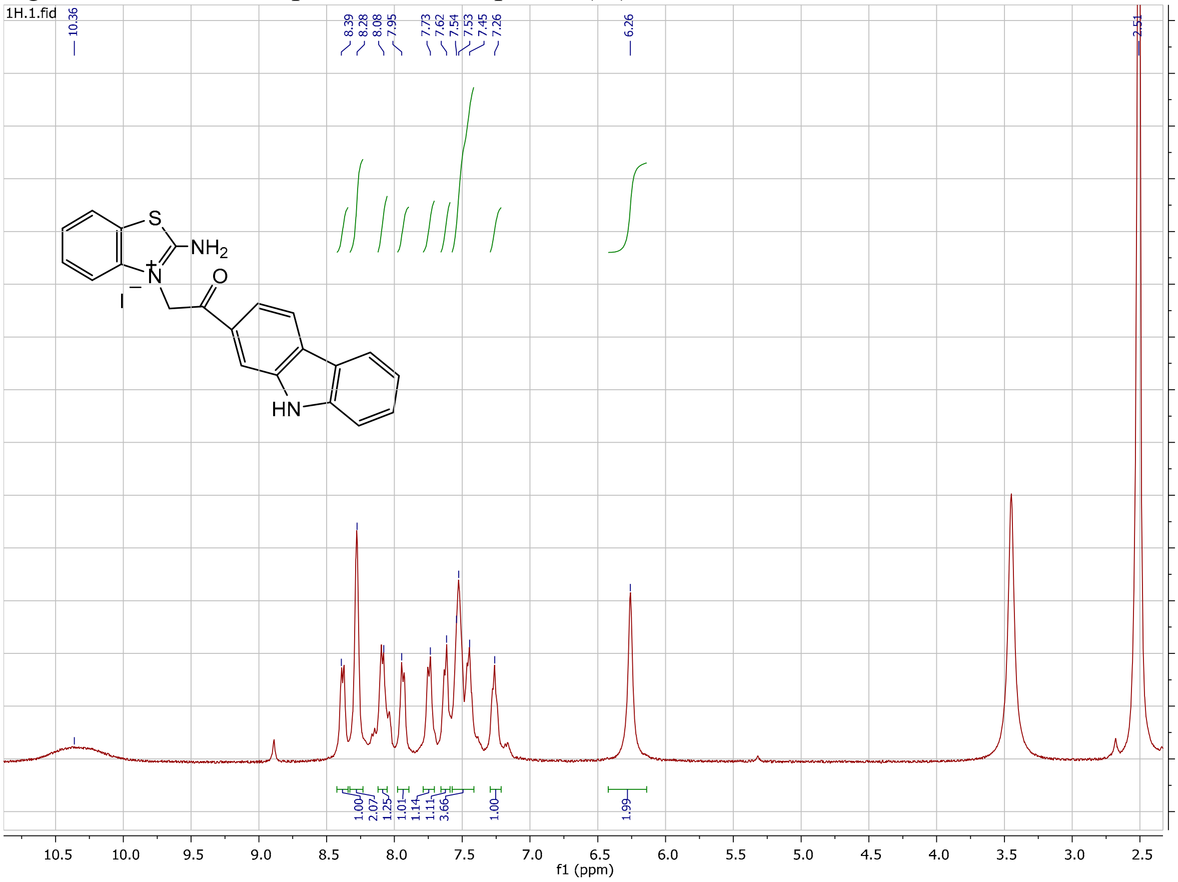

Figure S12. ^{13}C NMR spectrum of compound (3f).

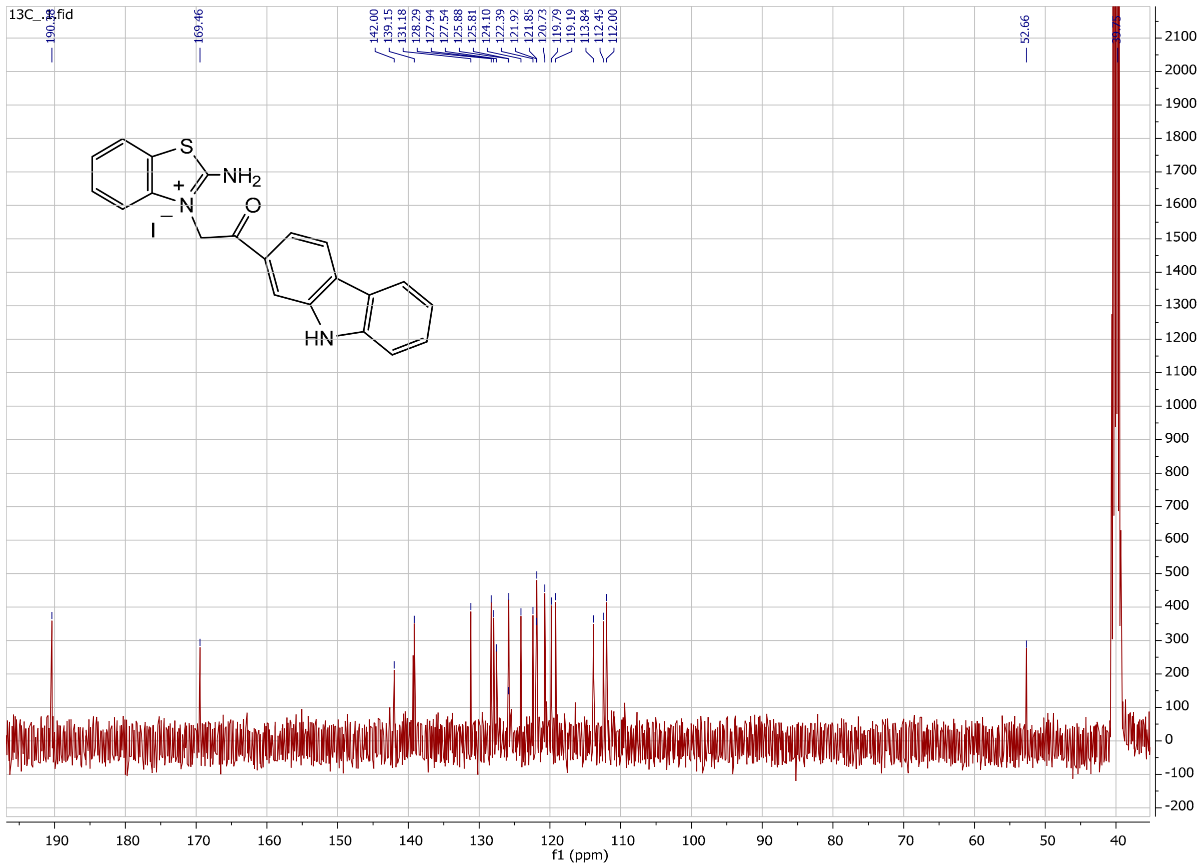

Figure S13. ^{1}H NMR spectrum of compound (6a).

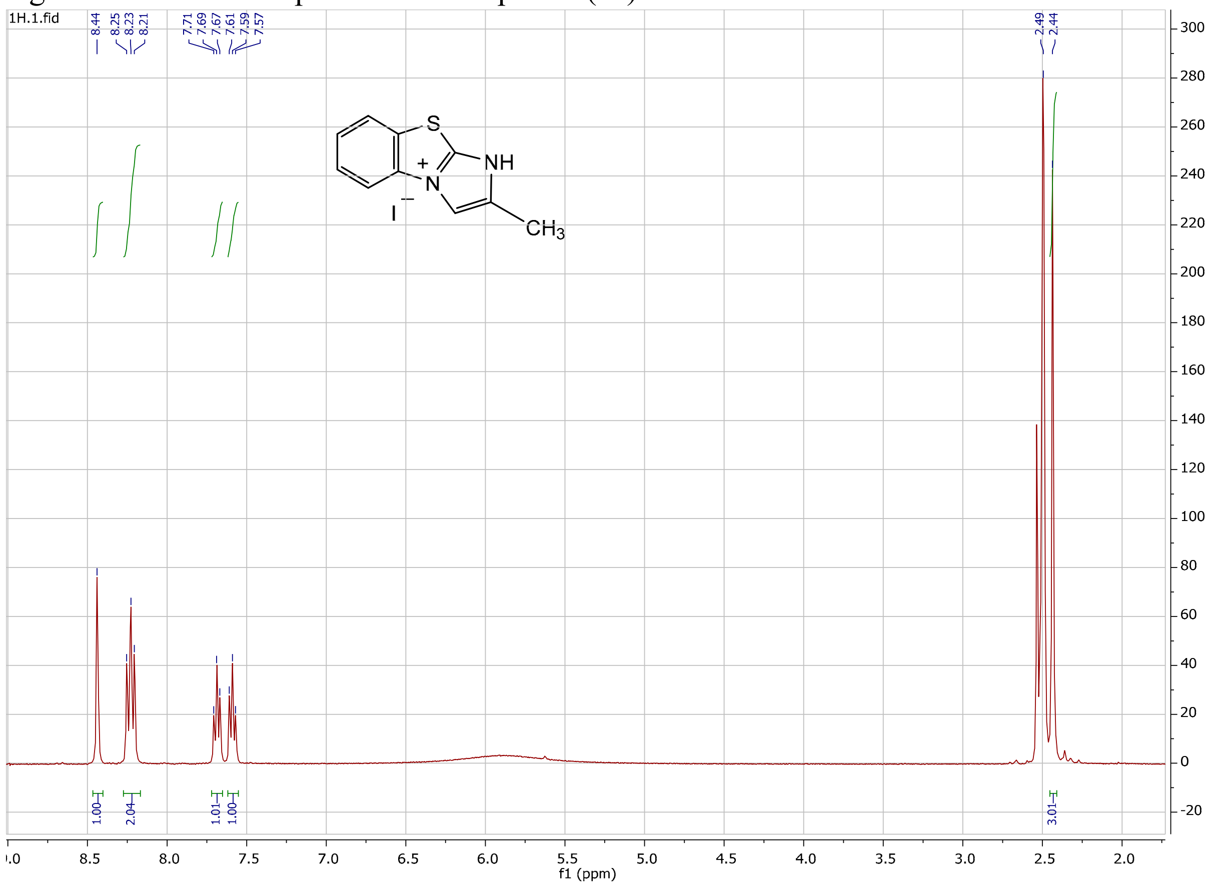

Figure S14. ^{13}C NMR spectrum of compound (6a).

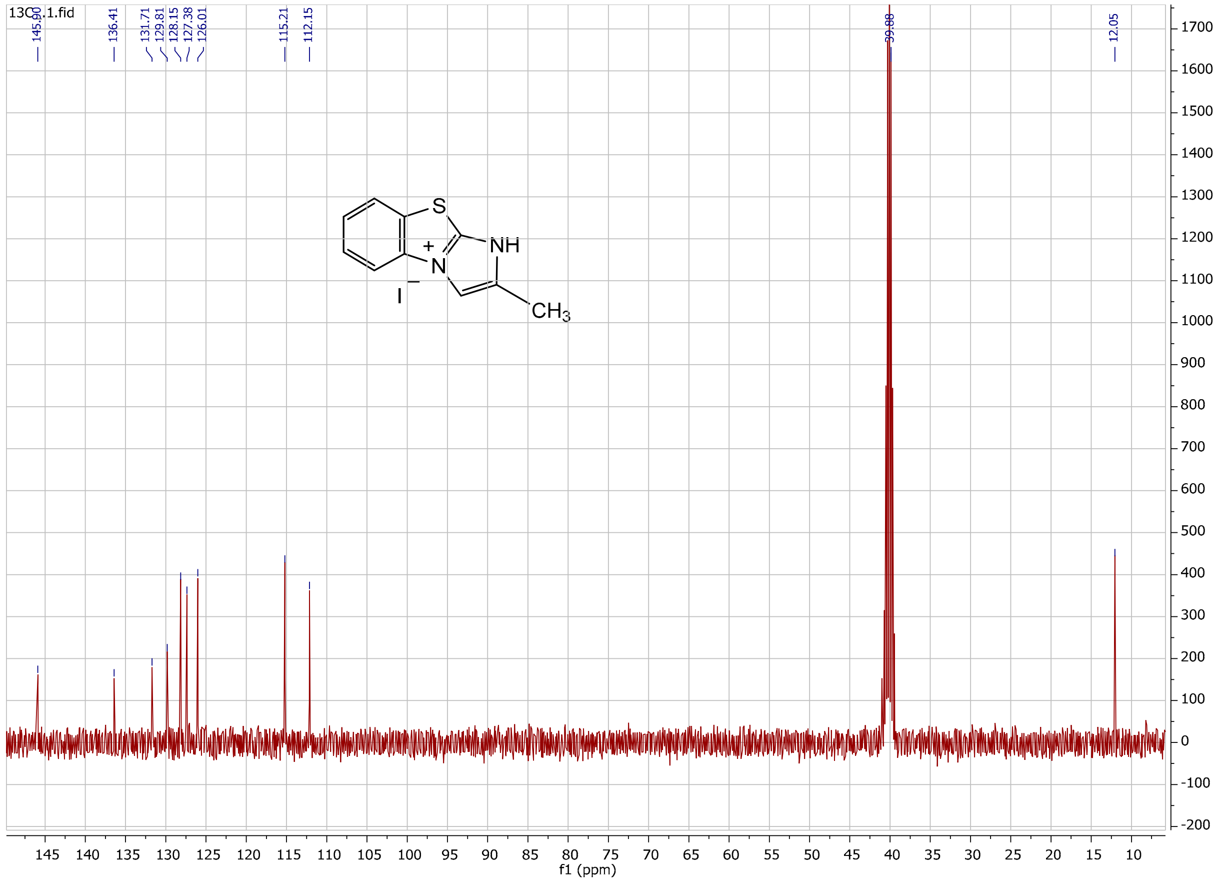

Figure S15. ^{1}H NMR spectrum of compound (6b).

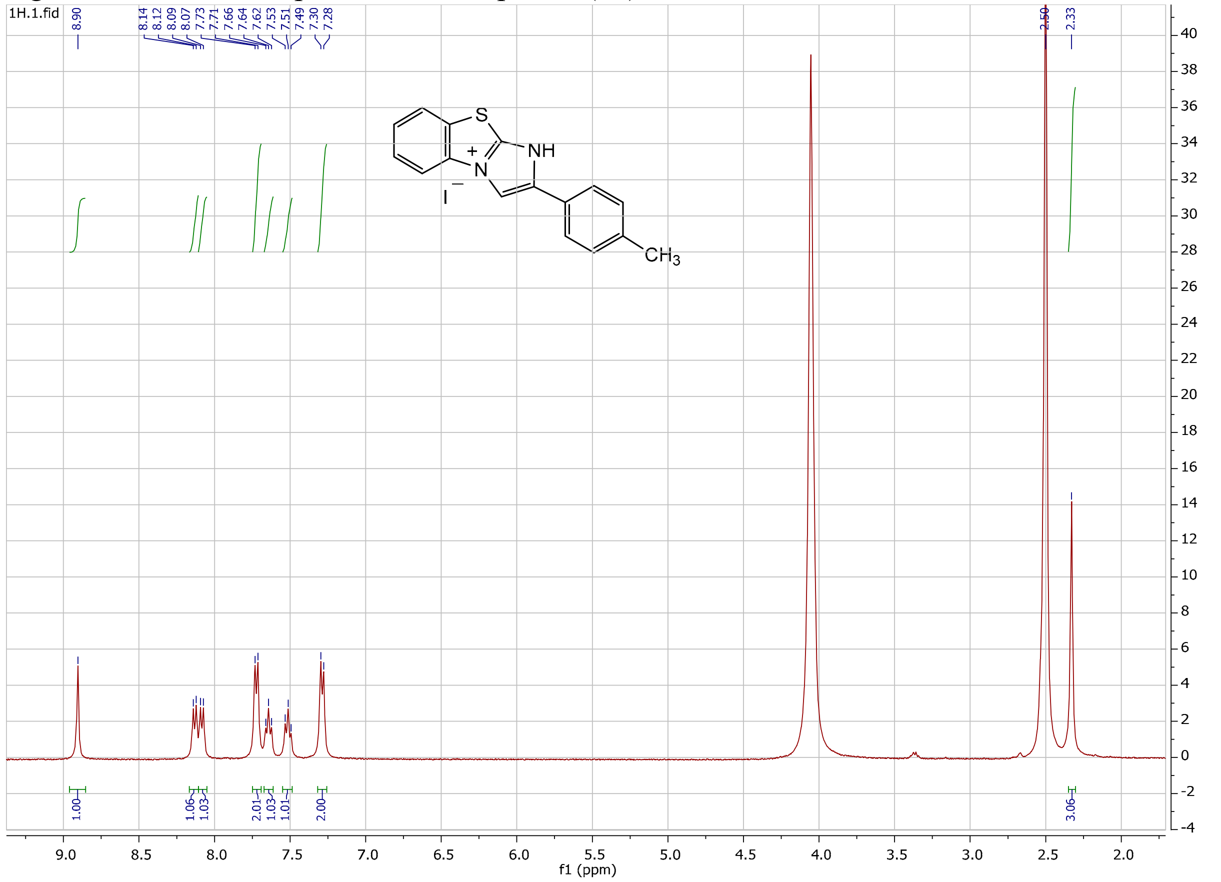

Figure S16. ^{13}C NMR spectrum of compound (6b).

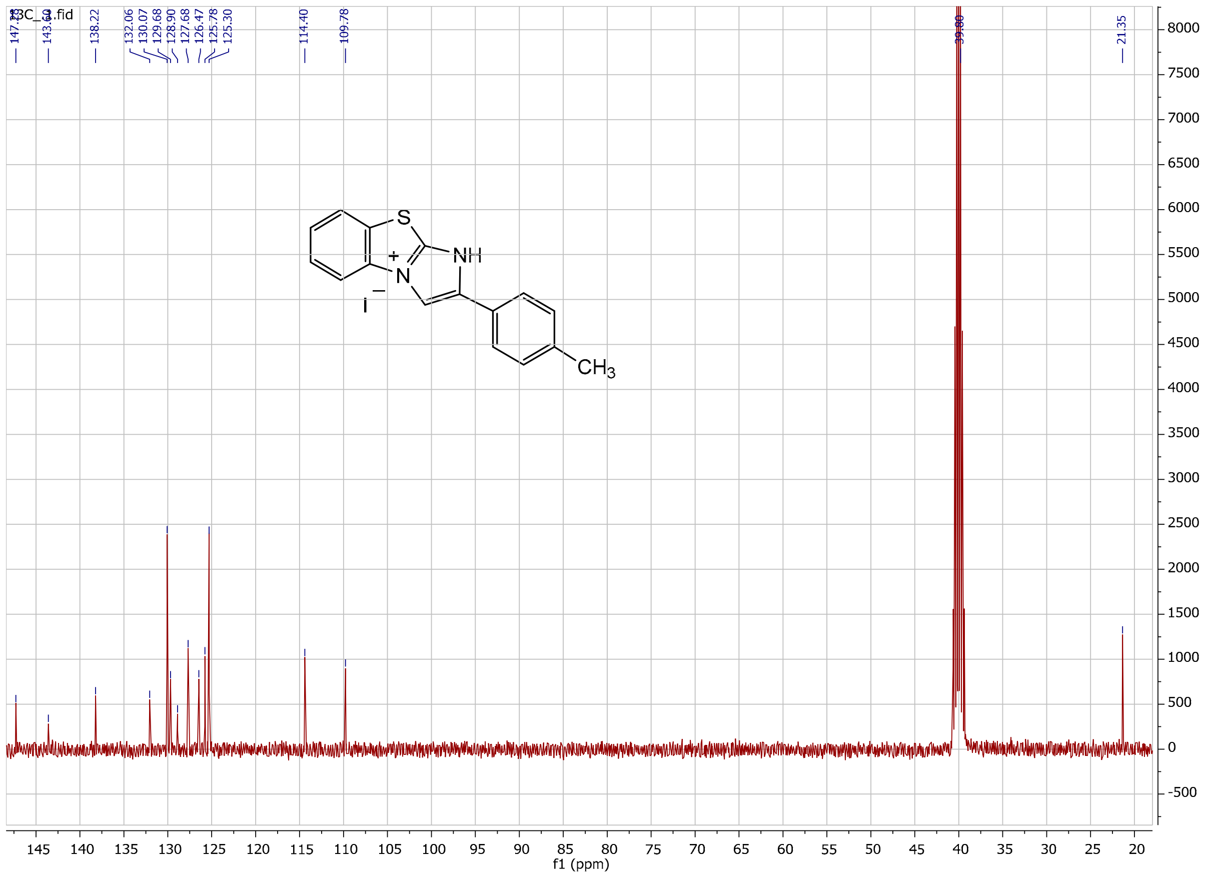

Figure S17. ^{1}H NMR spectrum of compound (6c).

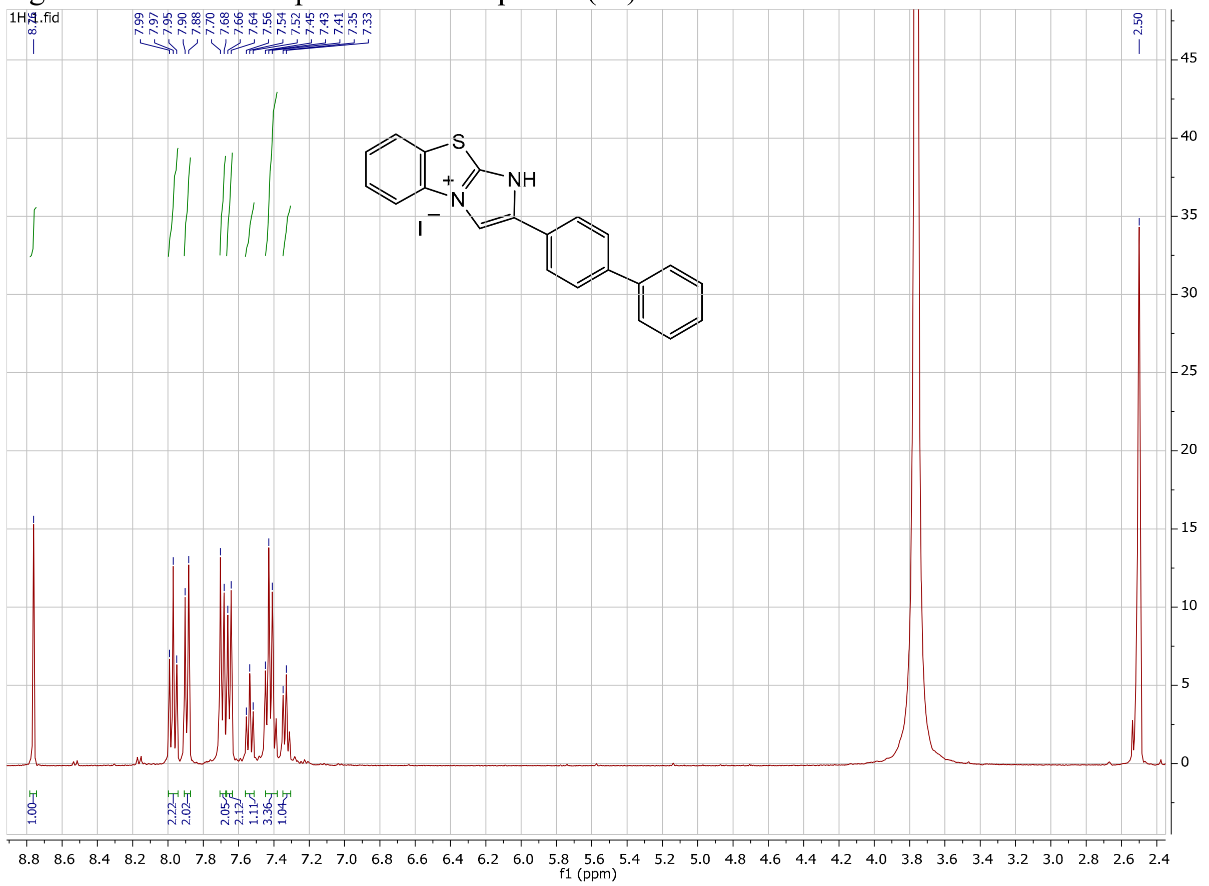

Figure S18. ^{13}C NMR spectrum of compound (6c).

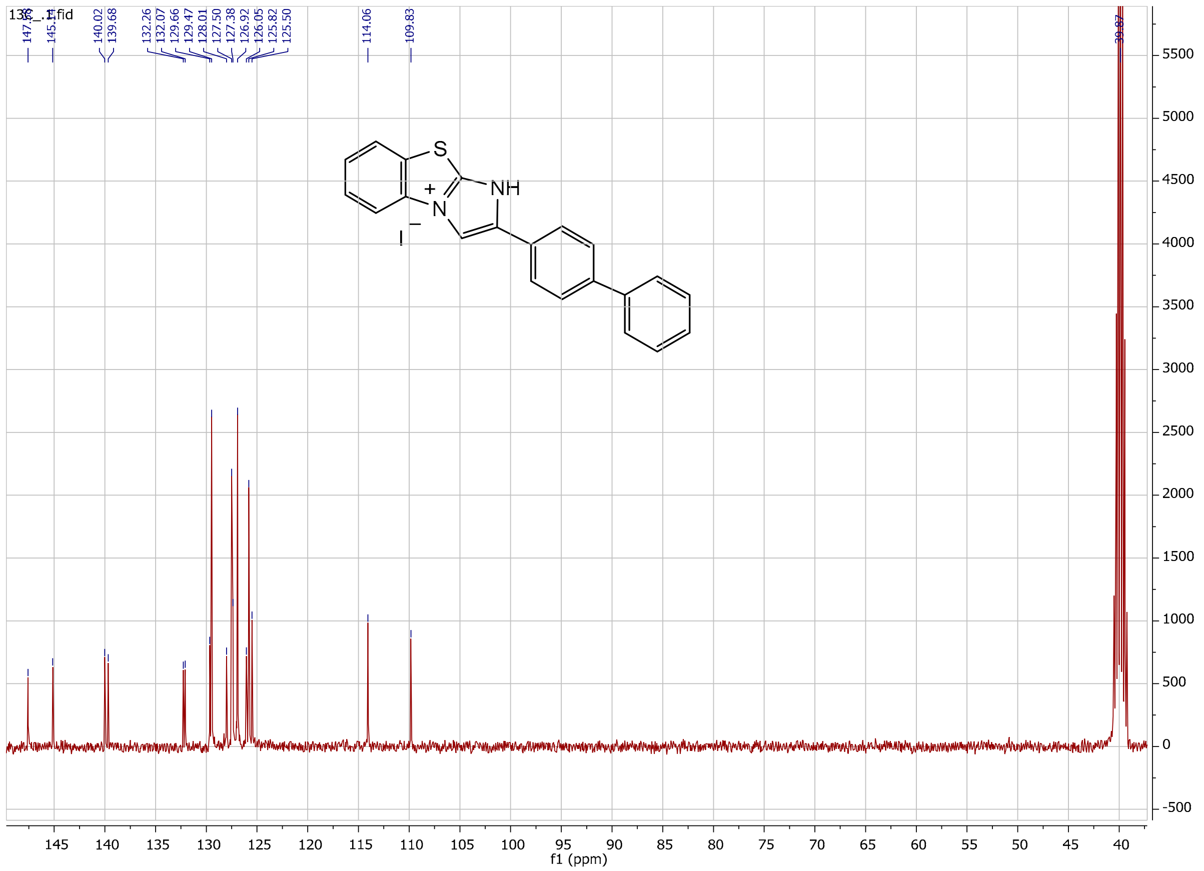

Figure S19. ^{1}H NMR spectrum of compound (6d).

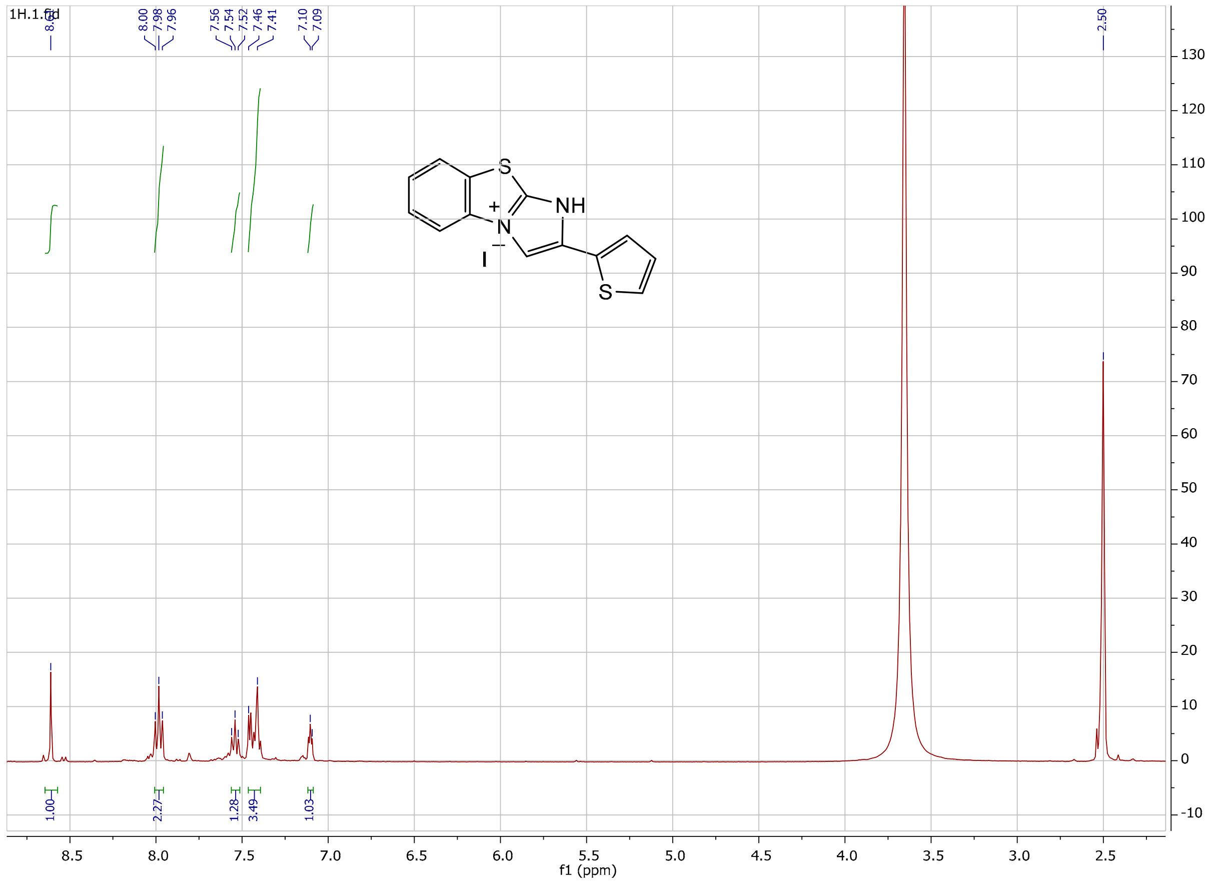

Figure S20. ^{13}C NMR spectrum of compound (6d).

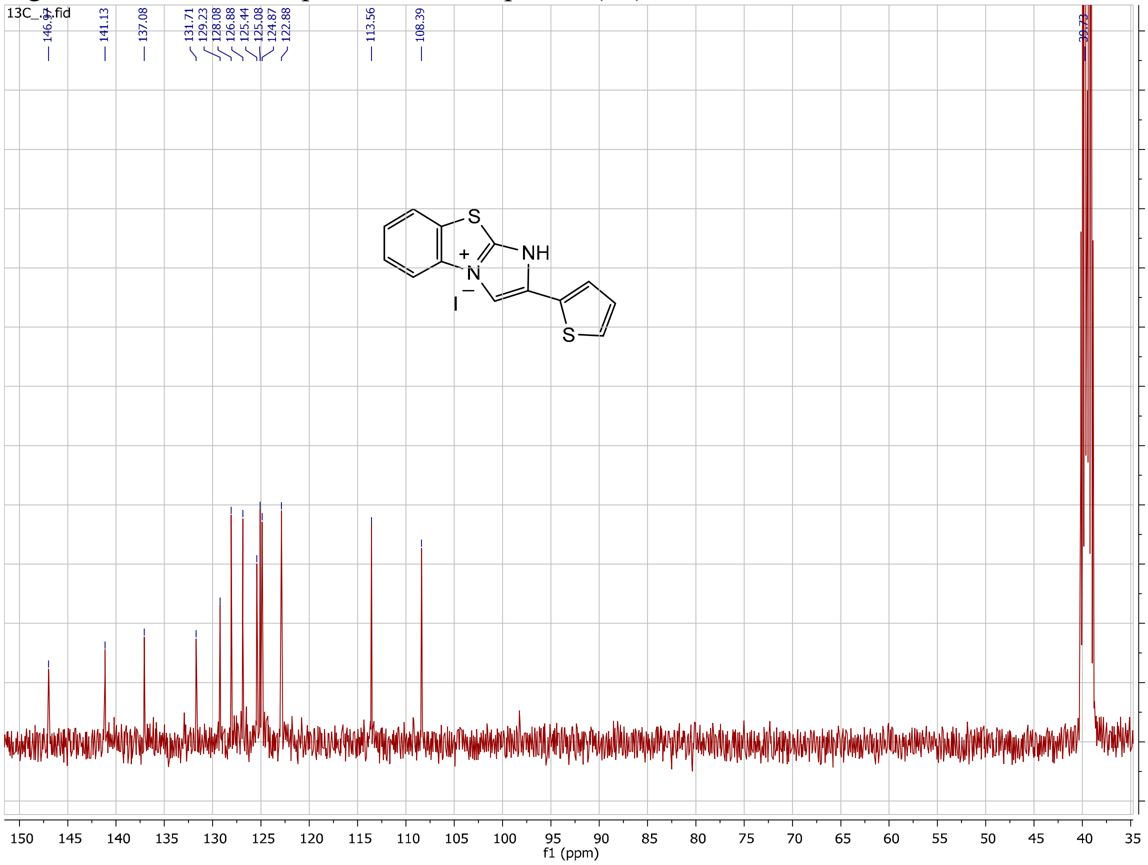

Figure S21. ^{1}H NMR spectrum of compound (6e).

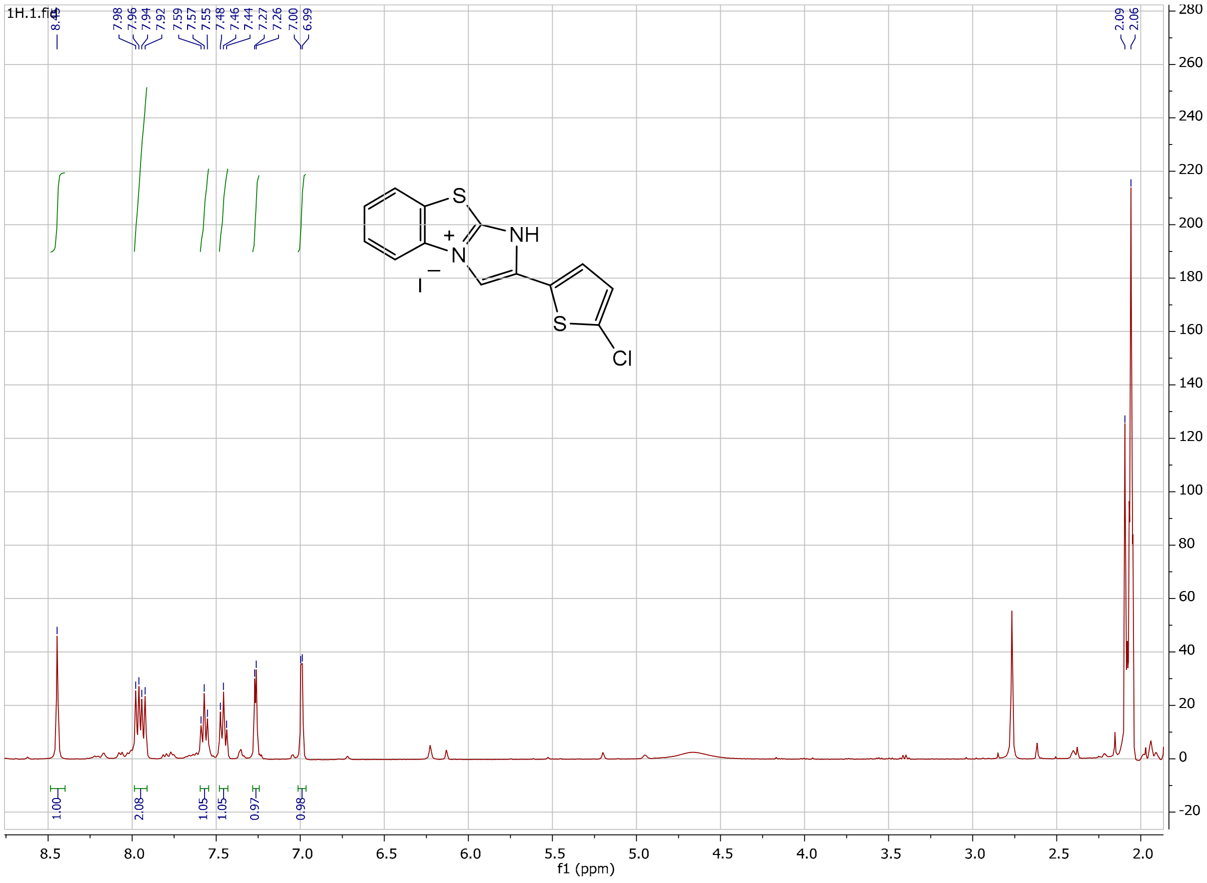

Figure S22. ^{13}C NMR spectrum of compound (6e).

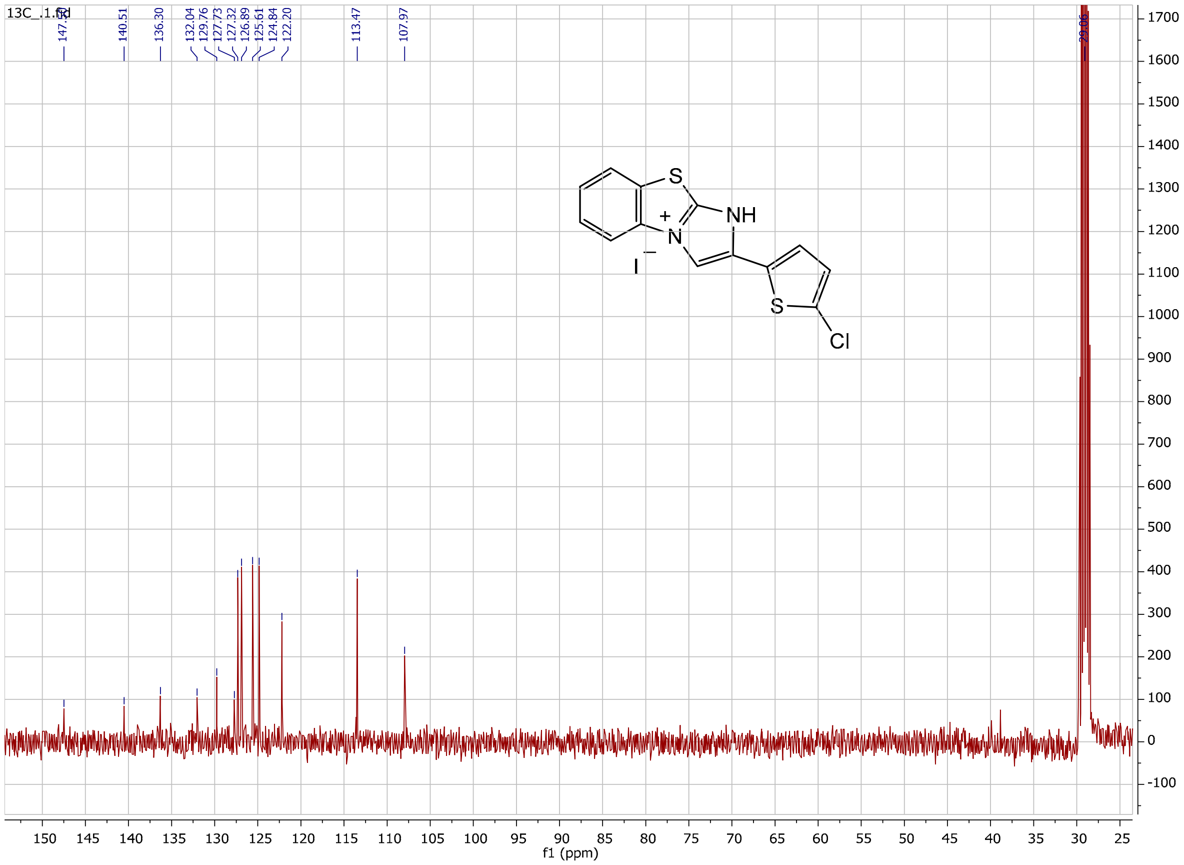

Figure S23. ^{1}H NMR spectrum of compound (6f).

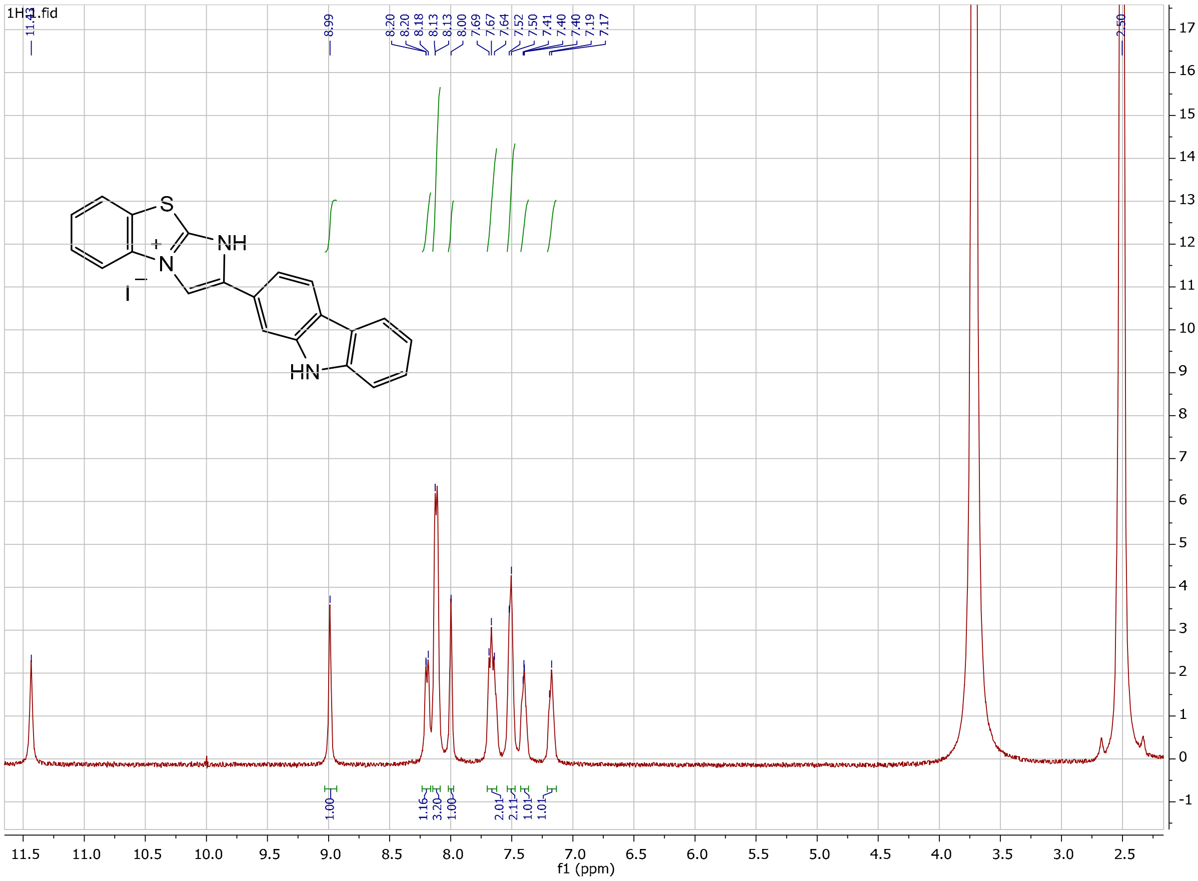

Figure S24. ^{13}C NMR spectrum of compound (6f).

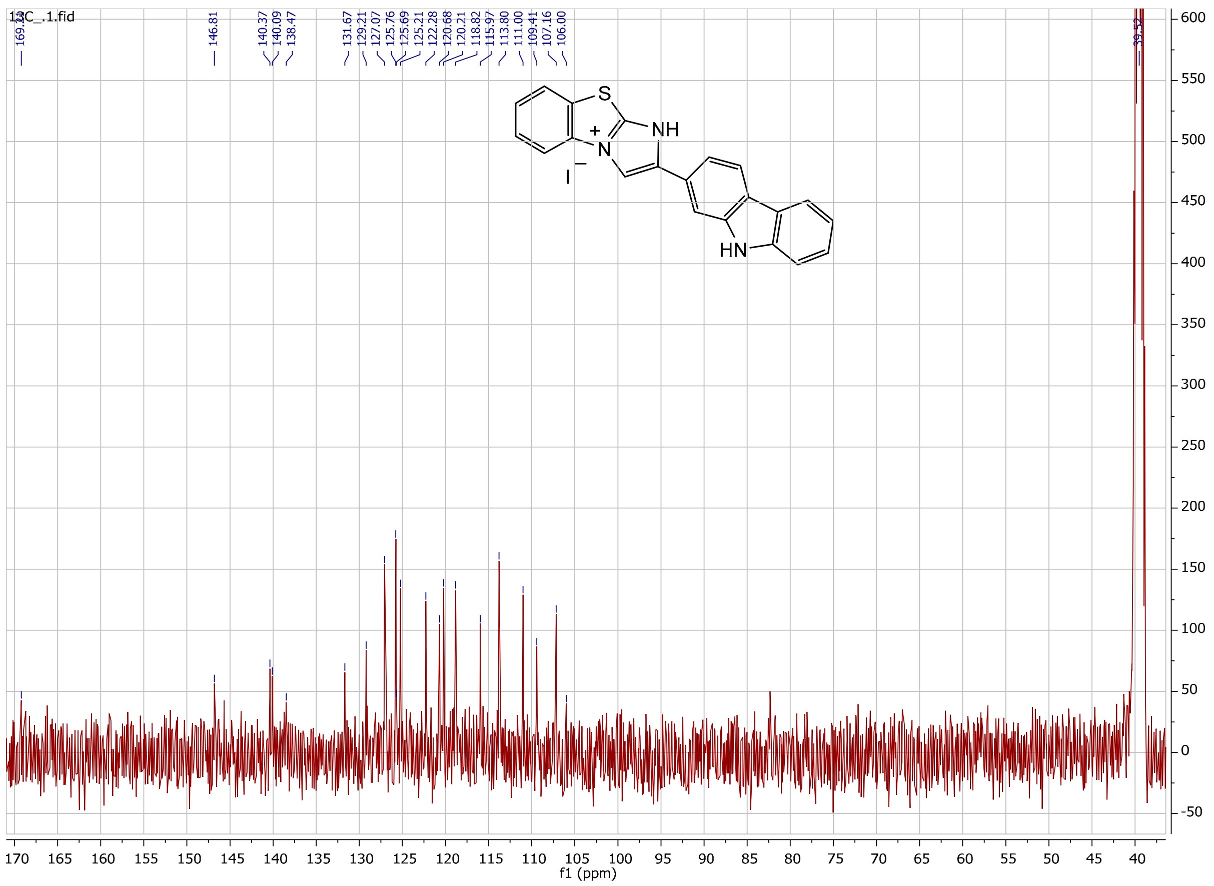

Figure S25. ^{1}H NMR spectrum of compound (4d).

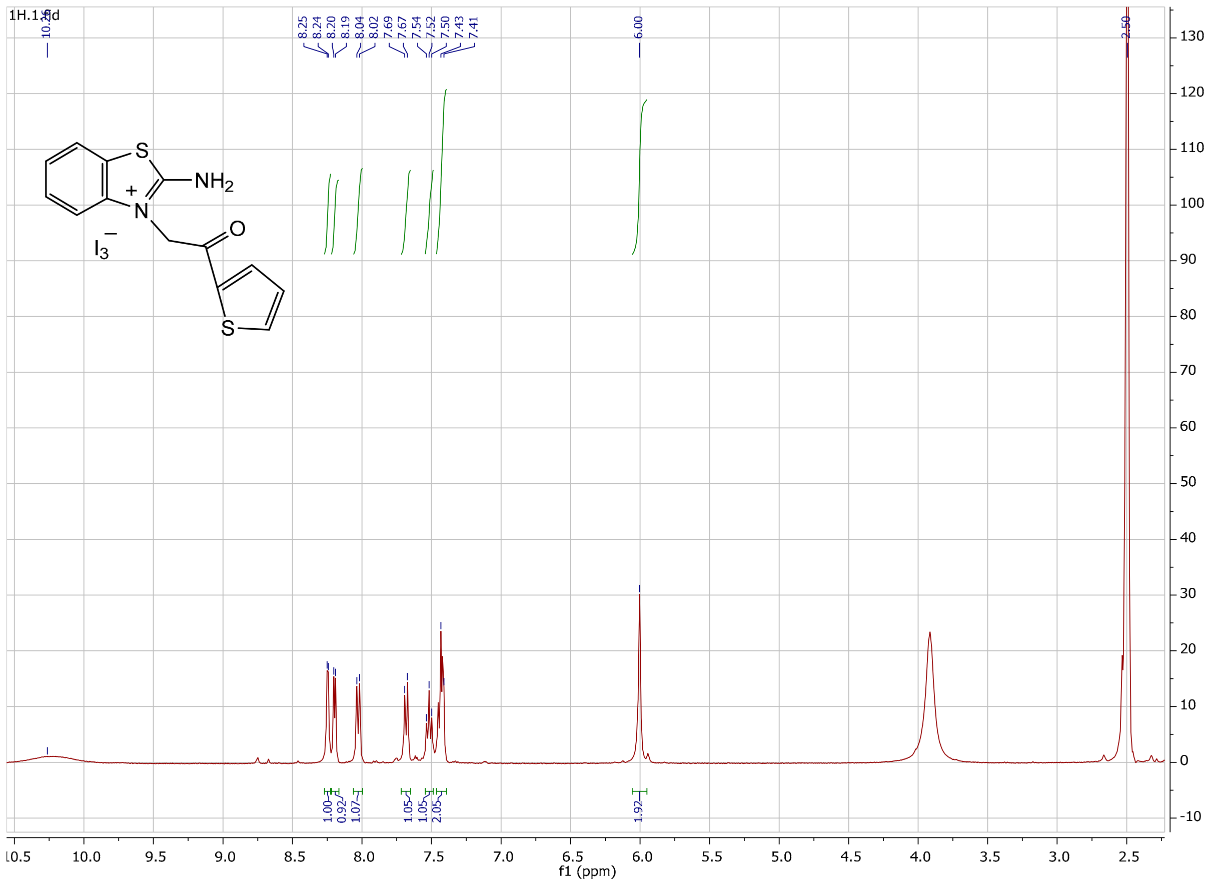

Figure S26. ^{13}C NMR spectrum of compound (4d).

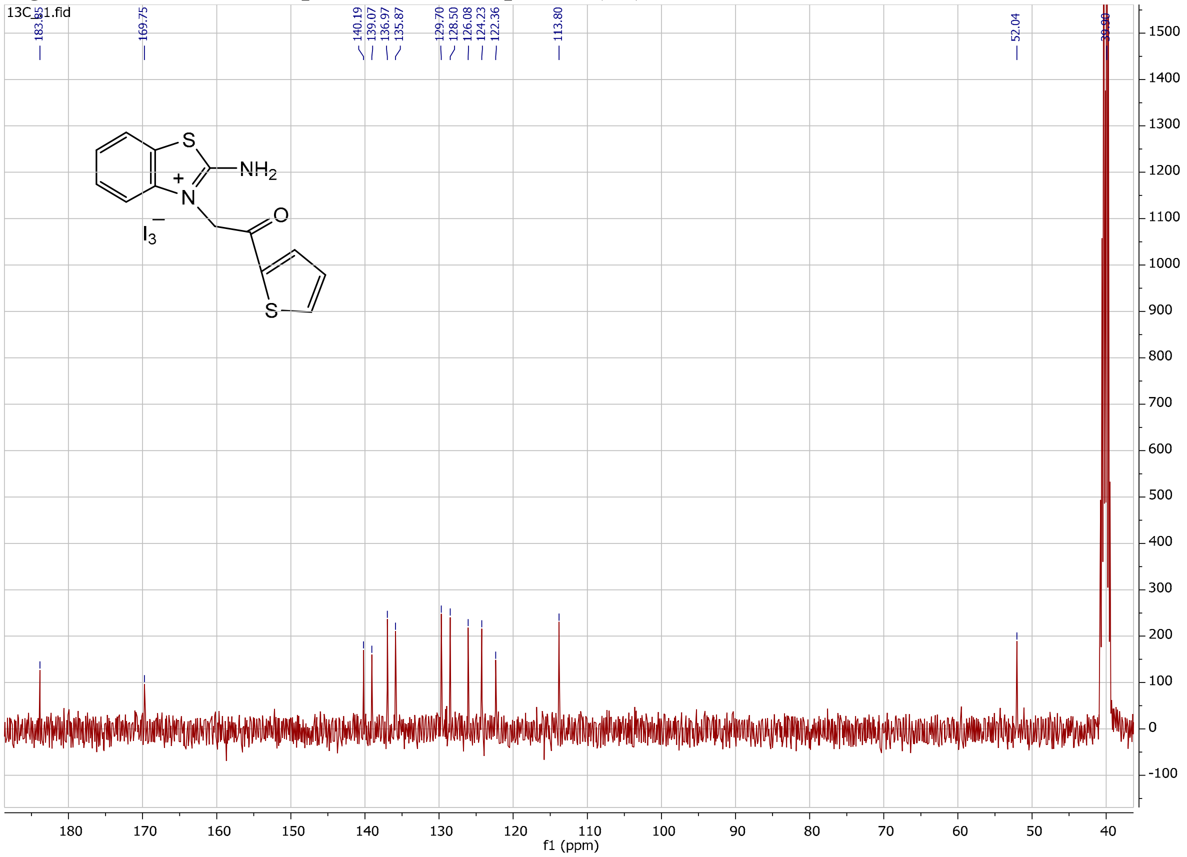

Figure S27. ^{1}H NMR spectrum of compound (4e).

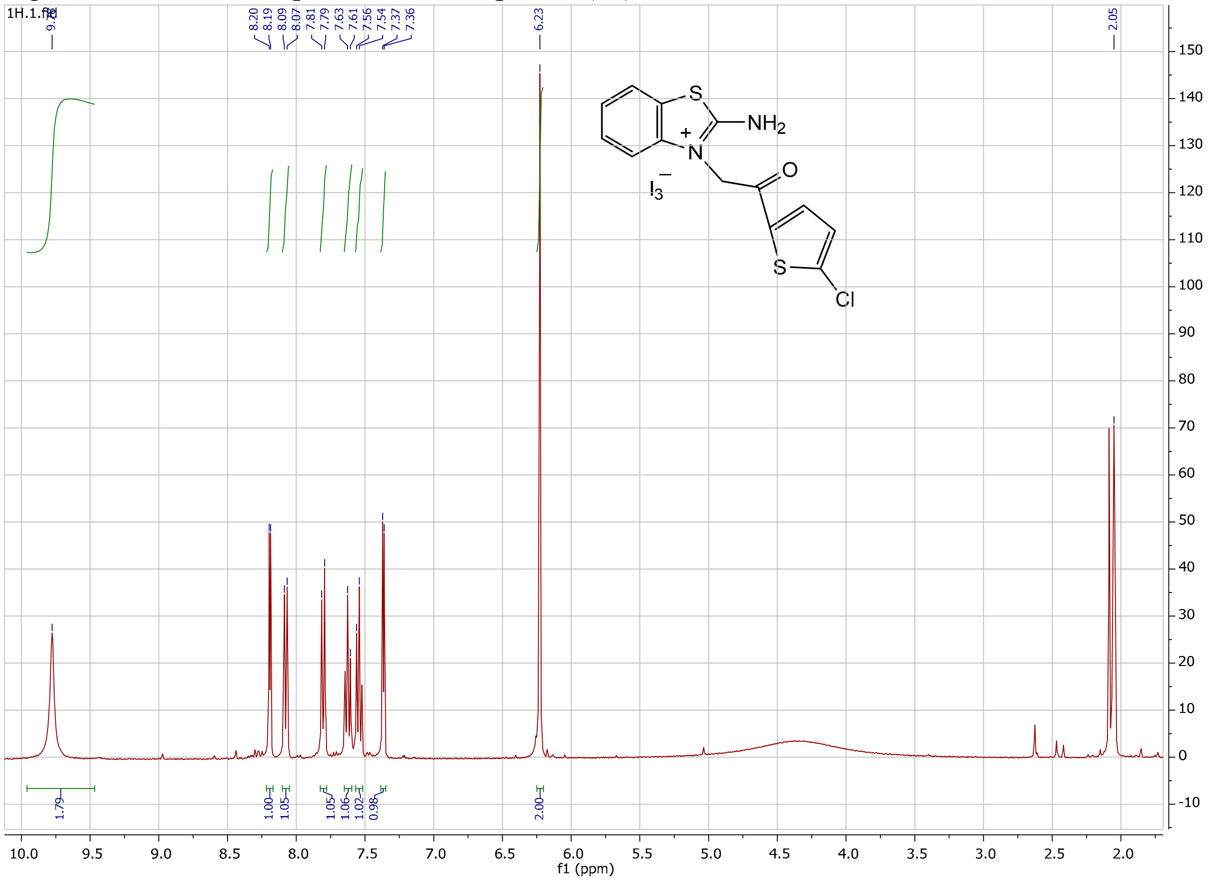

Figure S28. ^{13}C NMR spectrum of compound (4e).

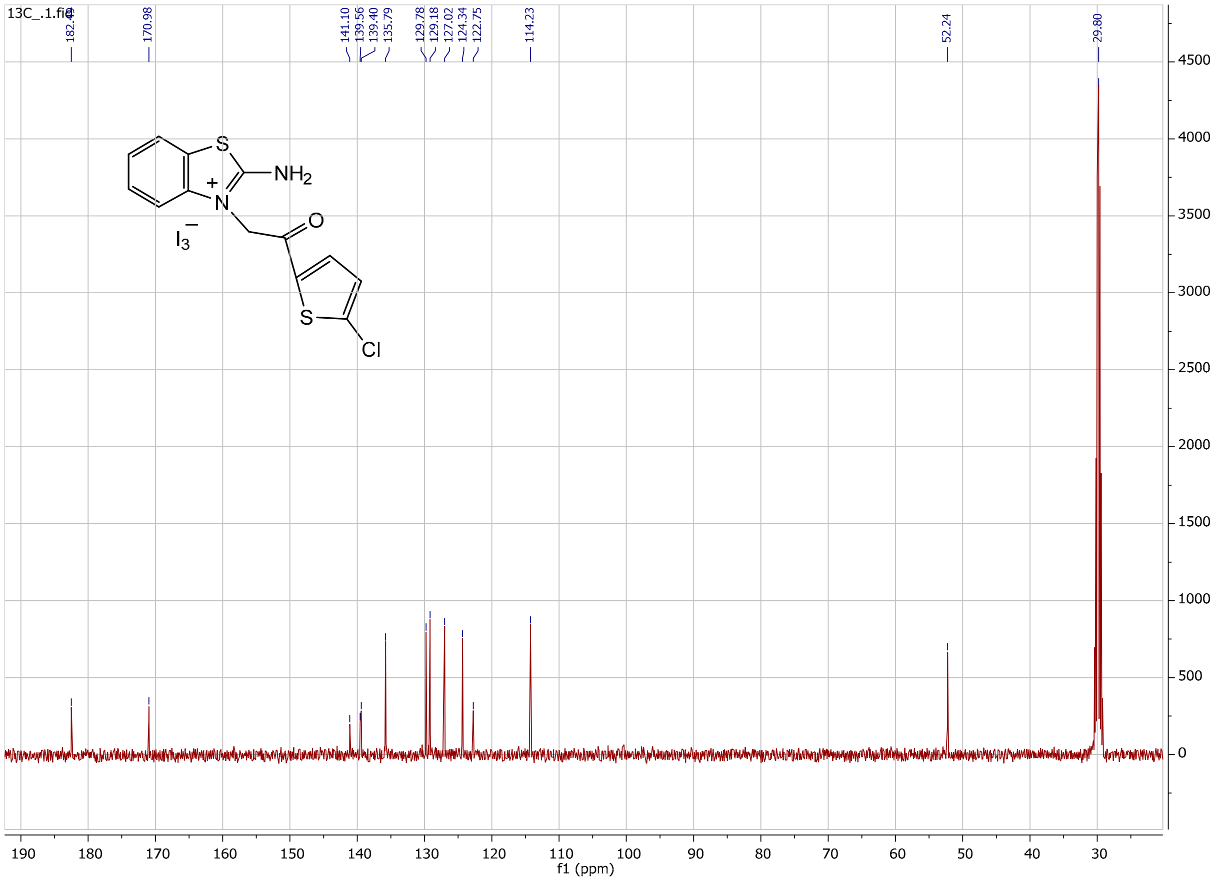

Figure S29. ^{1}H NMR spectrum of compound (6a).

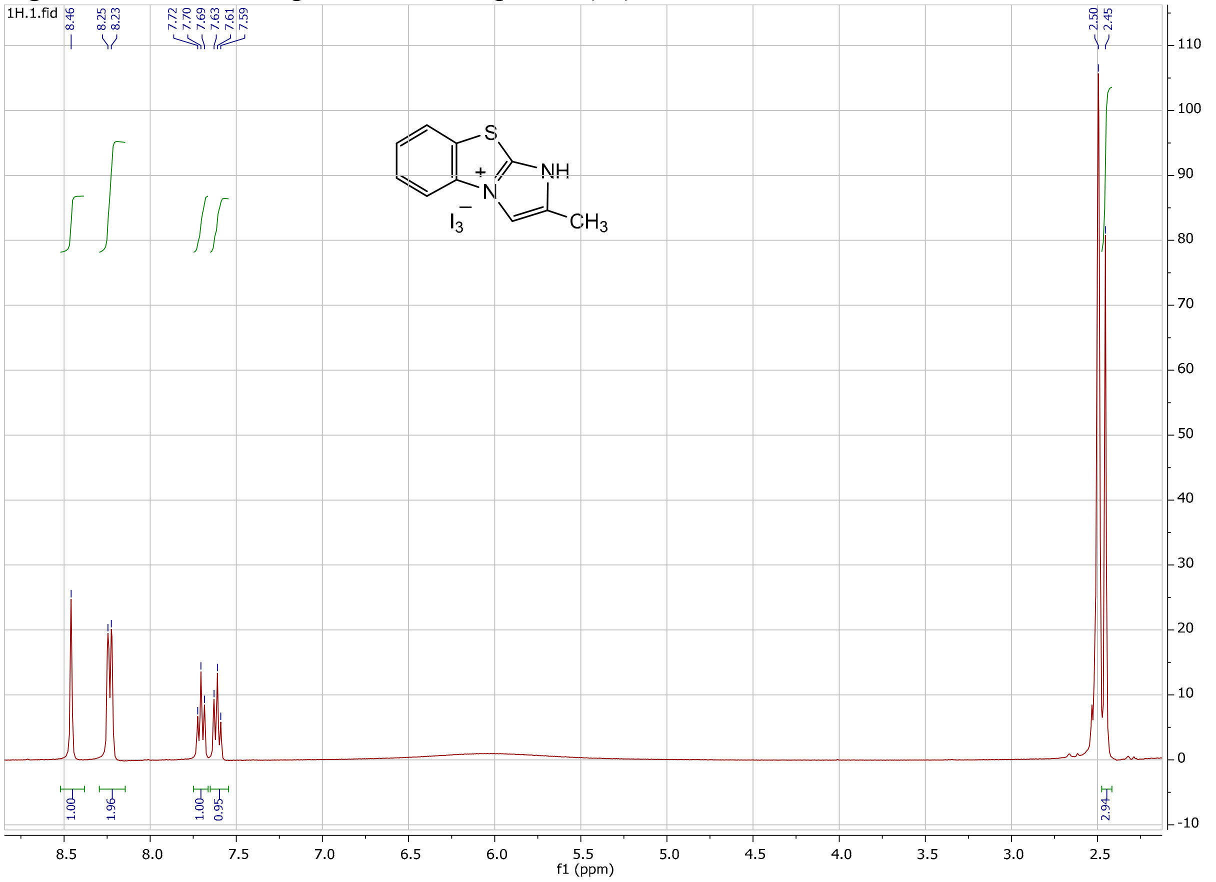

Figure S30. ^{13}C NMR spectrum of compound (6a).

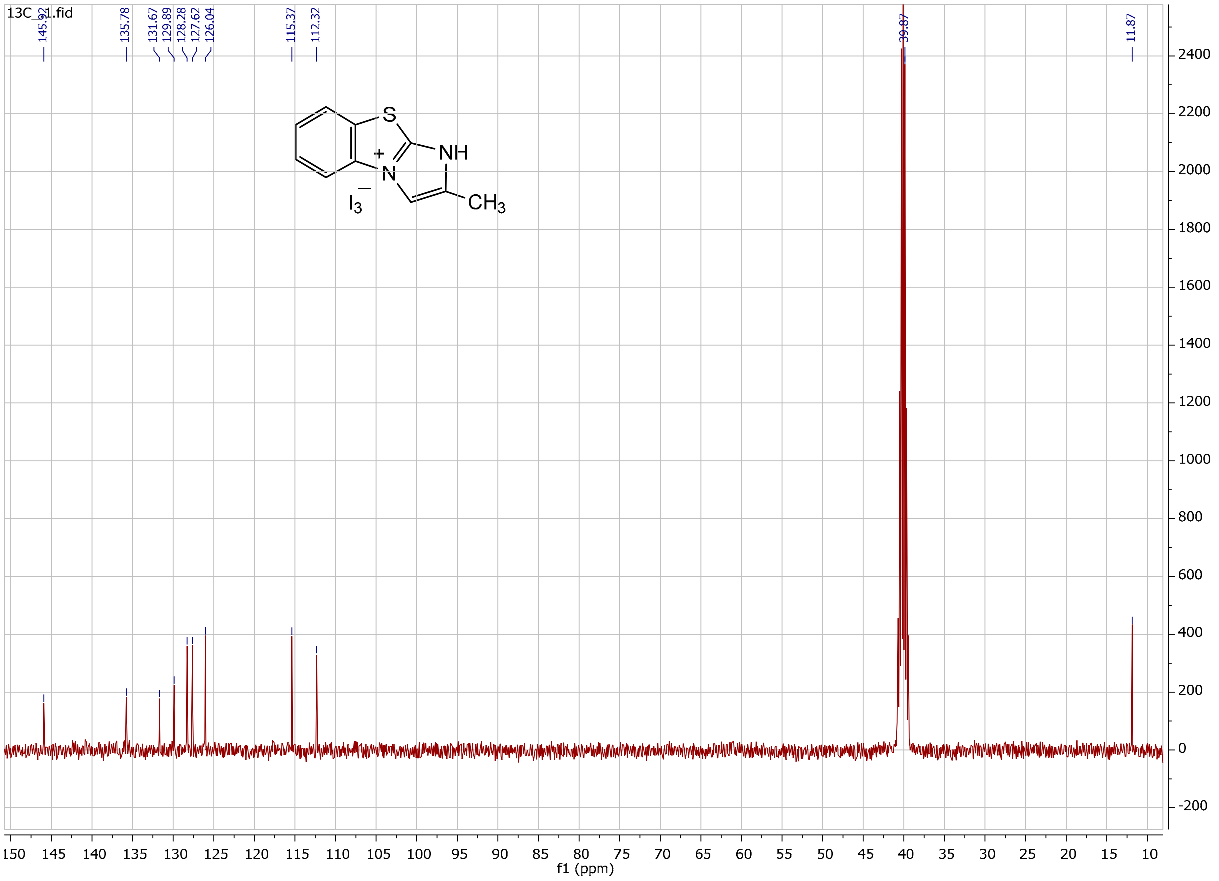

Figure S31. ¹H NMR spectrum of compound (6b).

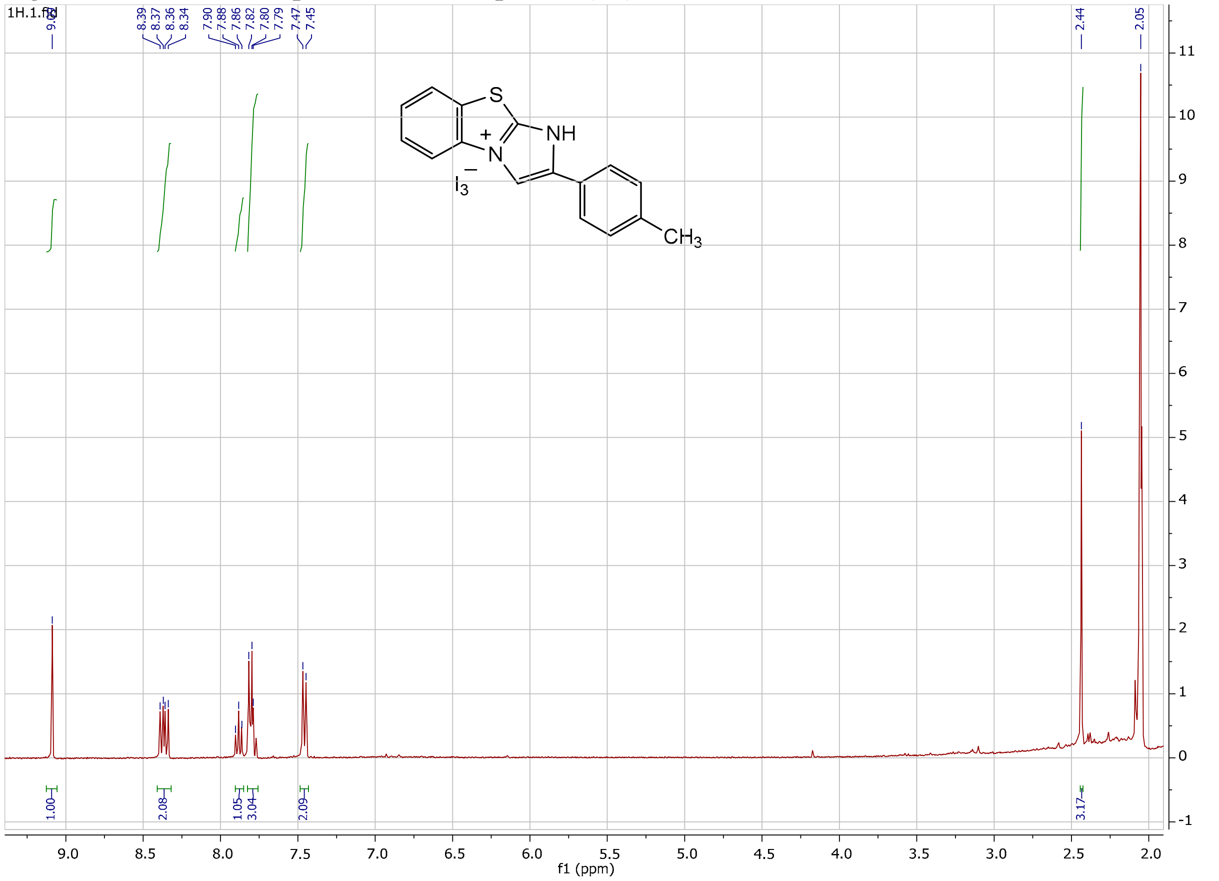

Figure S32. ¹³C NMR spectrum of compound (6b).

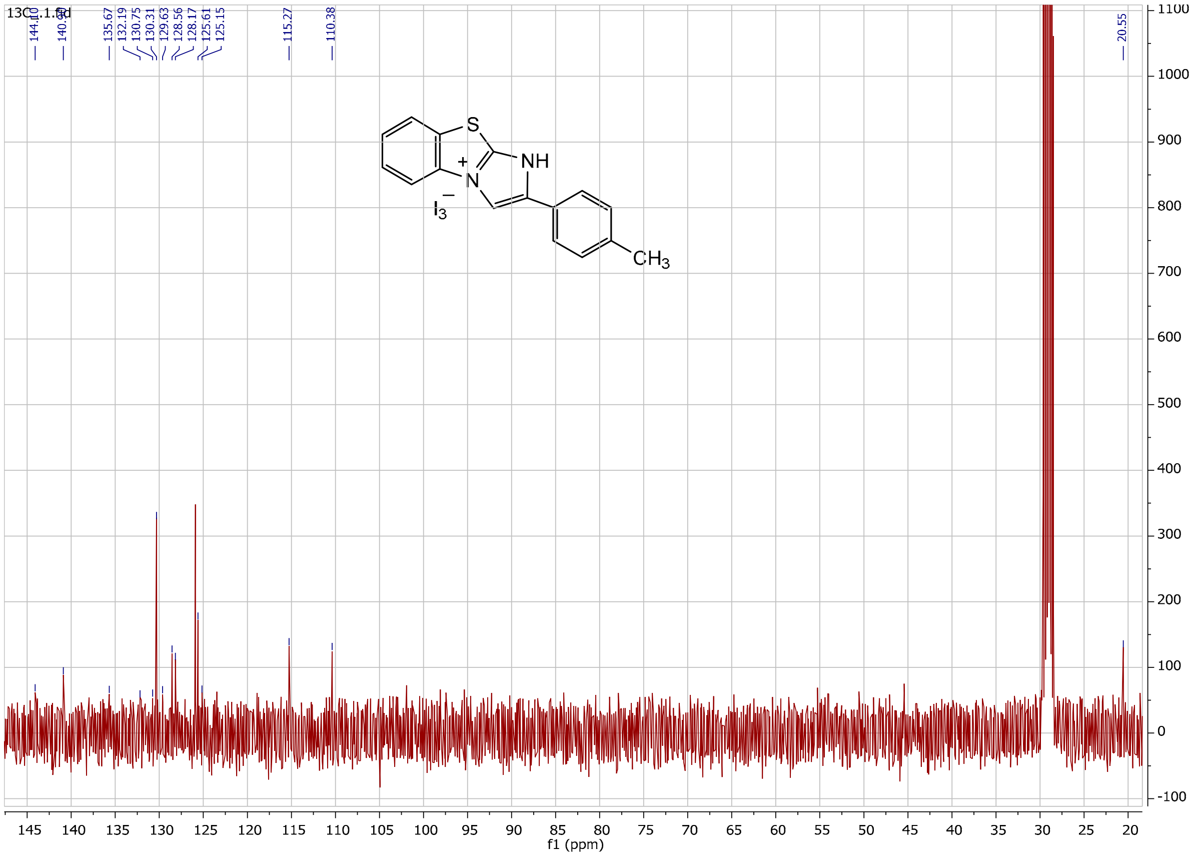

Figure S33. ^{1}H NMR spectrum of compound (6c).

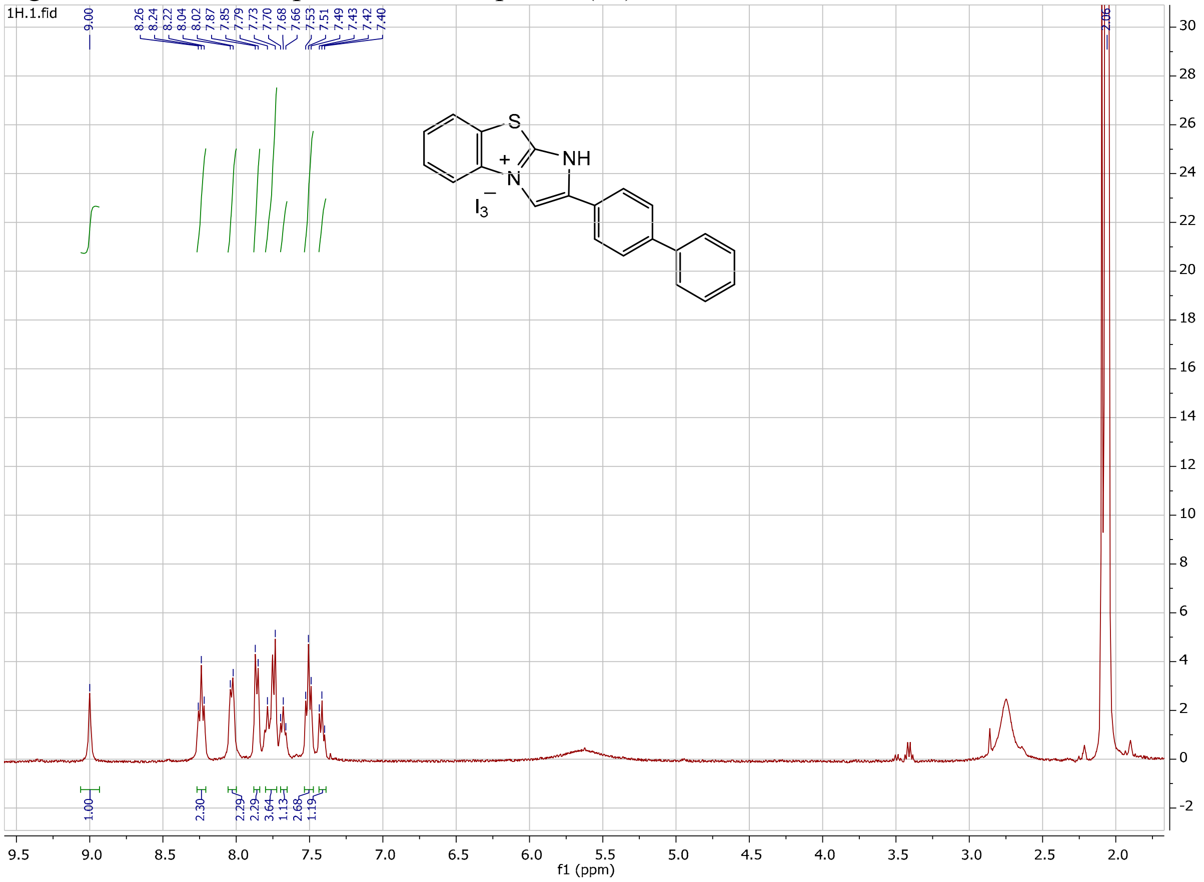

Figure S34. ^{13}C NMR spectrum of compound (6c).

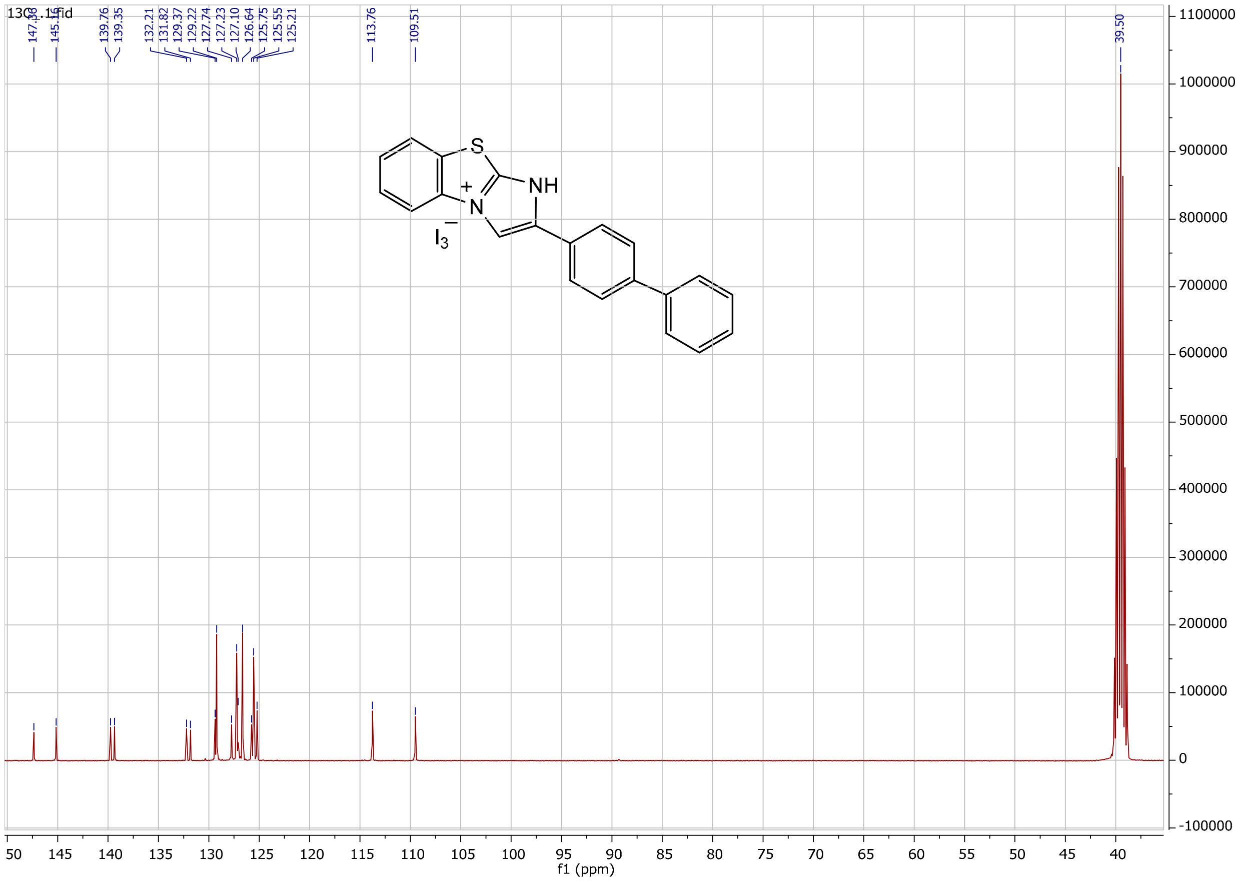

Figure S35. ^{1}H NMR spectrum of compound (6d).

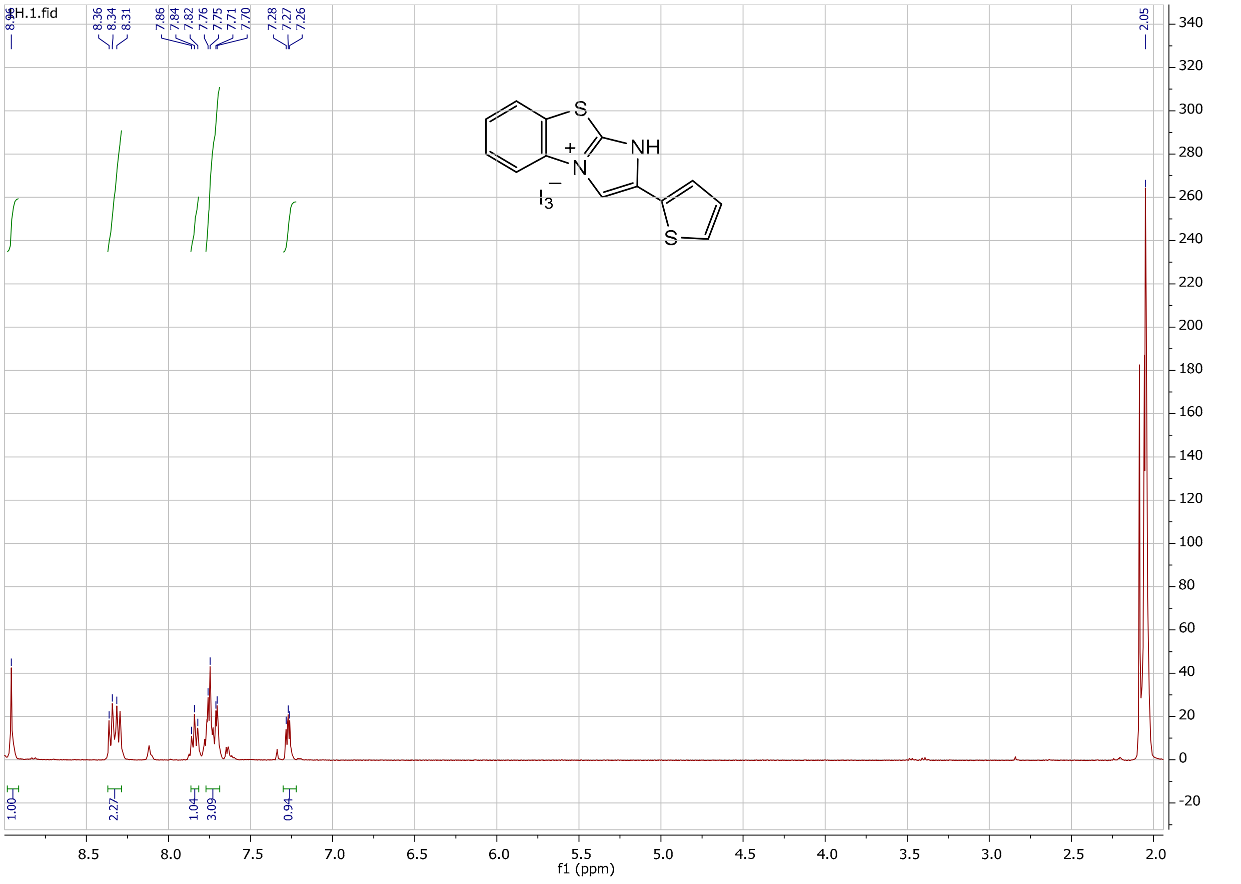

Figure S36. ^{13}C NMR spectrum of compound (6d).

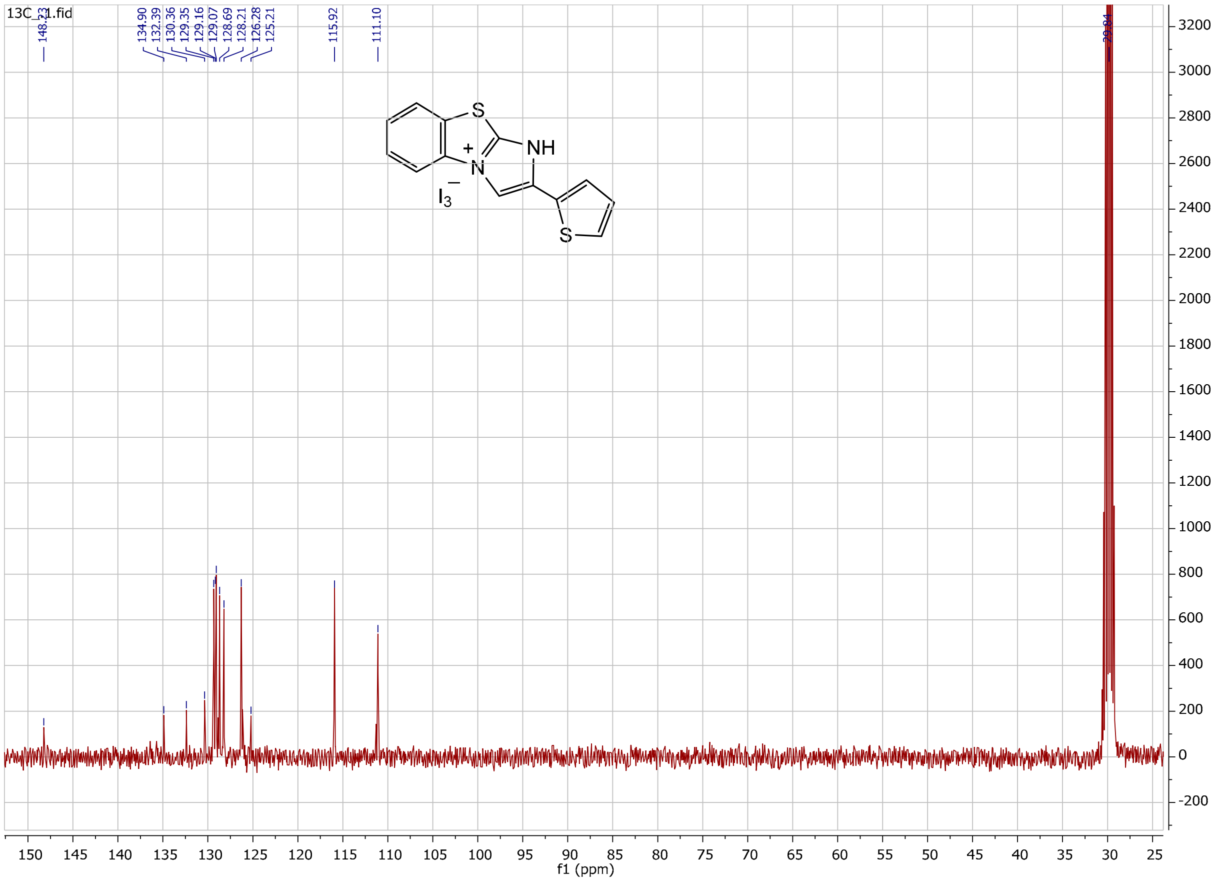

Figure S37. ^{1}H NMR spectrum of compound (6e).

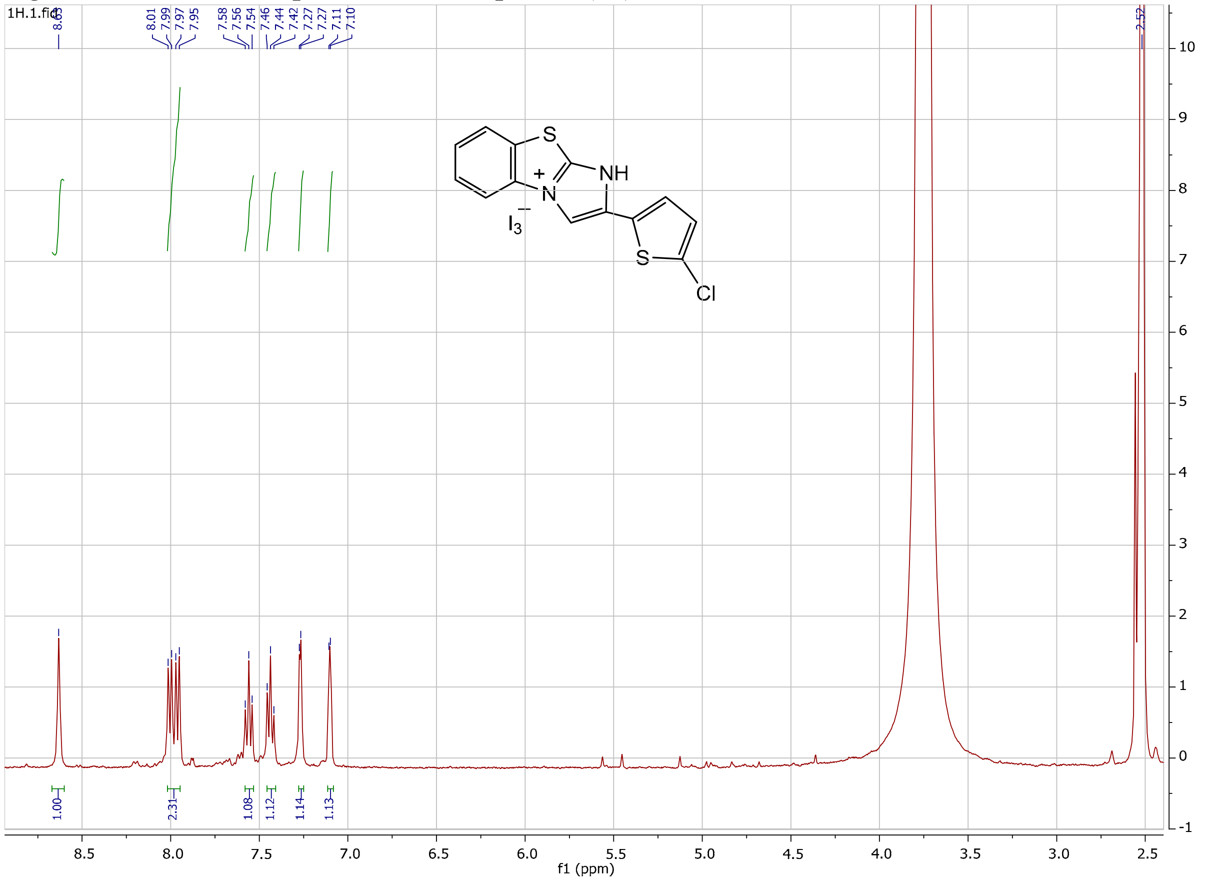

Figure S38. ^{13}C NMR spectrum of compound (6e).

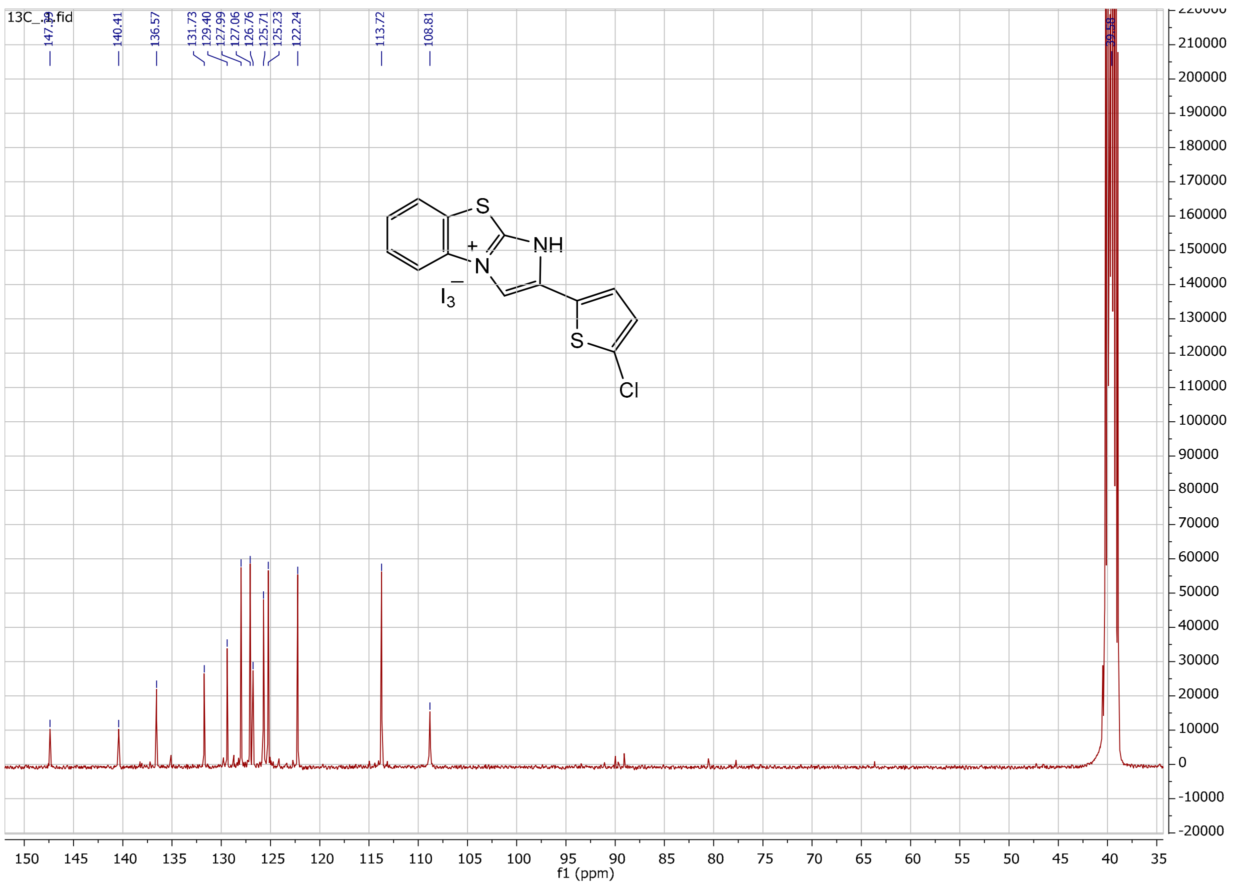

Figure S39. ^{1}H NMR spectrum of compound (6f).

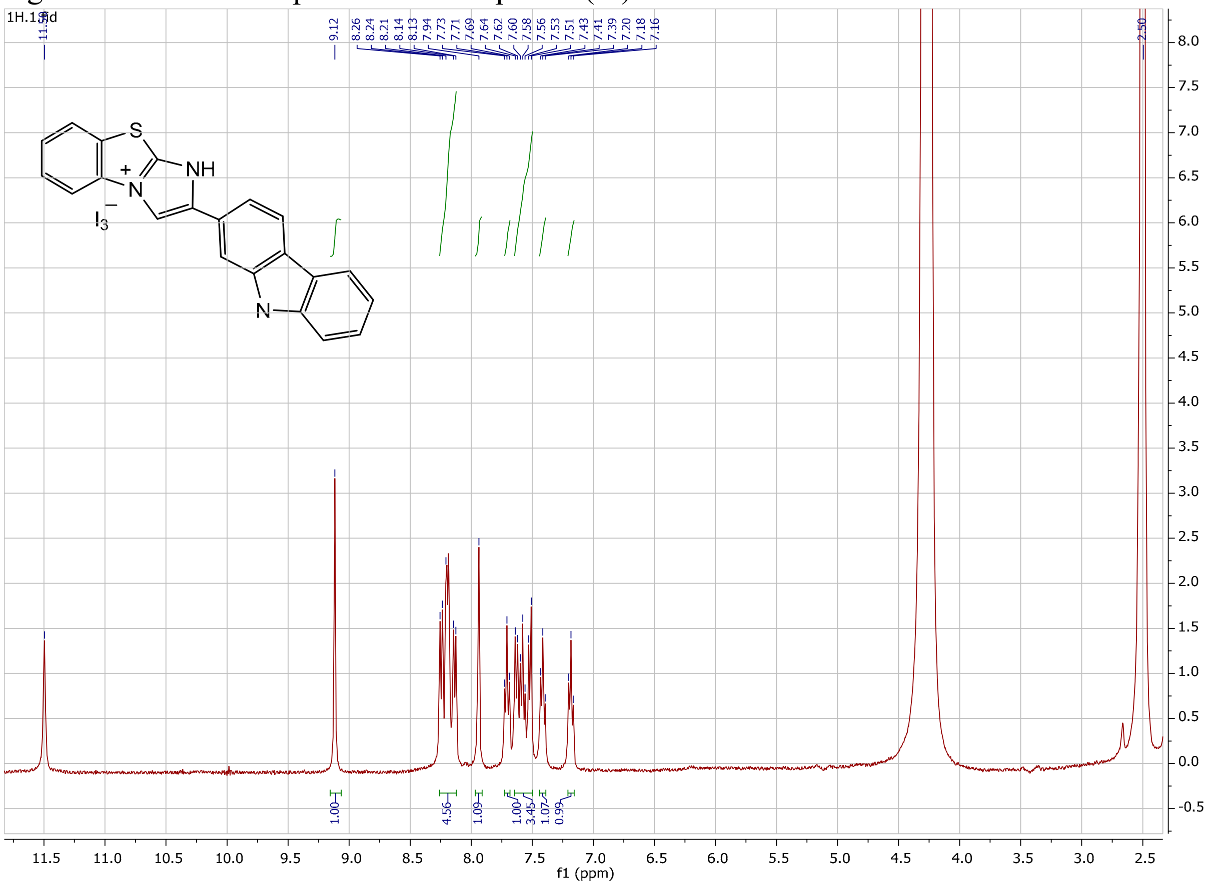

Figure S40. ^{13}C NMR spectrum of compound (6f).

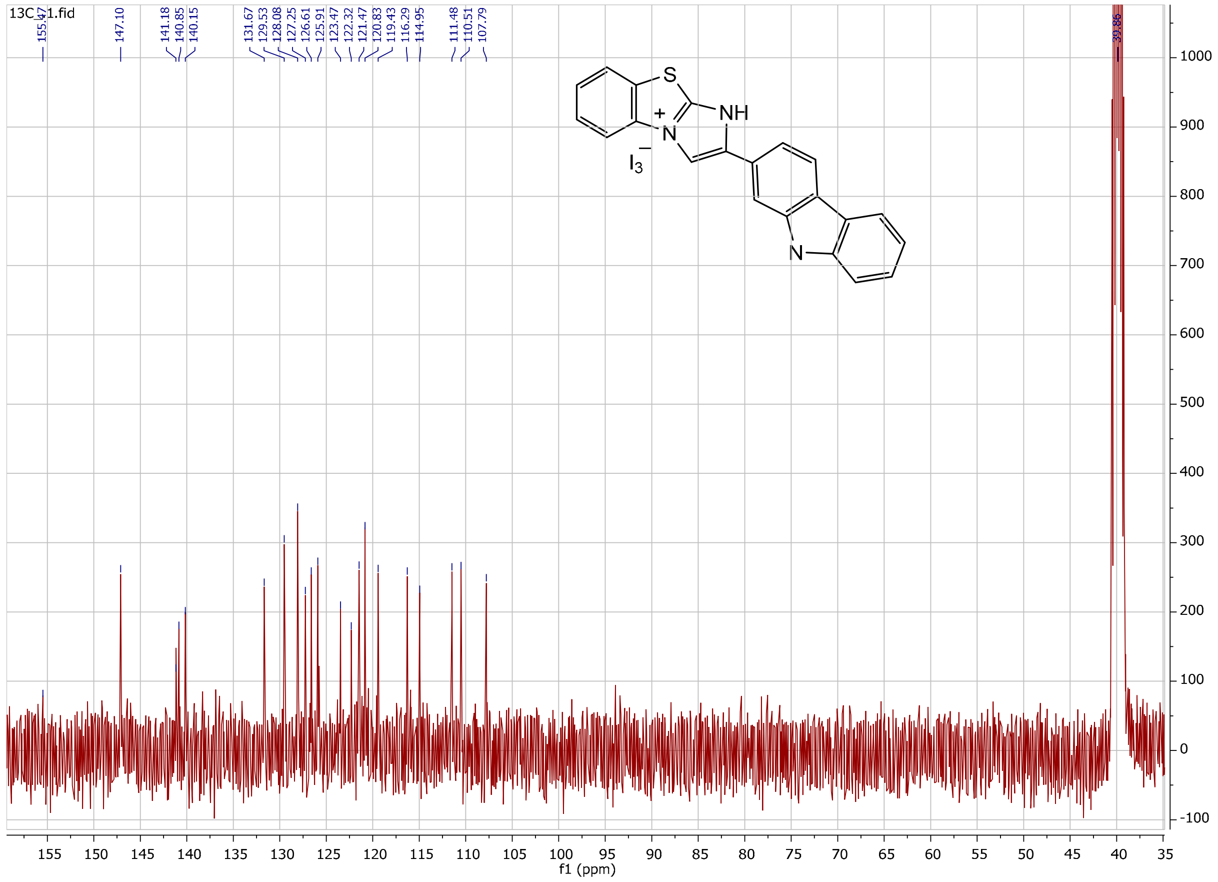
155.47
147.10
141.18
140.85
140.15
131.67
129.53
128.08
127.25
126.61
125.91
123.47
122.32
121.47
120.83
119.43
116.29
114.95
111.48
110.51
107.79
S
NH
N
+
I3
N
f1 (ppm)
